# Heat stress impacts affective processes and risk-taking behaviour

**DOI:** 10.1038/s41598-026-47250-x

**Published:** 2026-04-09

**Authors:** Jannic Wälde, Günter Amesberger, Sabine Würth, Markus Reichert, Thomas Finkenzeller

**Affiliations:** 1 Department of Sport and Exercise Science, University of Salzburg, Schloßallee 49, 5400 Hallein, Austria; 2 Department of Psychiatry and Psychotherapy, Central Institute of Mental Health, Medical Faculty Mannheim, Heidelberg University, Mannheim, Germany

**Keywords:** Health care, Physiology, Psychology, Psychology

## Abstract

The understanding of heat stress on human behaviour is critical due to increasing frequency of extreme heat events. Heat stress has been shown to detrimentally alter affective states and cognition, effects that are particularly critical for individuals exposed to heat conditions such as firefighters, medical personnel, and athletes. Therefore, this study examined how heat stress, exercise, and wearing firefighter protective gear interact to influence affect and decision-making. A sample of 36 healthy young males was assigned to either a passive, combined or heat-stress-free group. Subjective perceptions of vitality, fatigue, thermal comfort and sensation were assessed, controlling for body core-, skin temperature, relative humidity in the jacket, and heart rate. Additionally, participants performed the Balloon Analogue Risk Task and the Beads Task. Linear mixed models revealed that passive heat stress increased fatigue, whereas vitality was not directly sensitive to heat stress. Fatigue and vitality bidirectionally influenced each other independent of heat stress, while thermal perception altered both affective states. Increased risk-taking behaviour was observed under prolonged combined heat stress. No effects on reflection impulsivity were found. Findings highlight differential impacts of heat stress on affective processes that can bias risk-taking behaviour. Particularly under combined heat stress, individuals may be prone to potentially riskier behaviour, insights that should be tested towards ecological validity in future proposals.

**Supplementary Information:**

The online version contains supplementary material available at 10.1038/s41598-026-47250-x.

## Introduction

Hot environmental temperatures challenge the thermoregulatory tolerance of humans in various domains of life^1^. High outdoor temperatures and heat waves occur more regularly, triggering various psychological and cognitive challenges^2^. Heat elicits negative affect, reduces emotional well-being, and increases fatigue while decreasing vitality^3–5^. Higher outdoor temperatures have been observed to impair cognitive function in verbal and math tests, and professional judges experience a decrease in decision-making consistency^6,7^.

Humans are especially susceptible to heat when performing physical work in hot environmental temperatures such as farming, construction work, sports, or emergency services^8–11^. Protective gear, such as that worn by firefighters, medical professionals and athletes, is essential for protecting against fire, infection and impact. However, it hinders eccrine sweat evaporation and therefore limits heat loss capacity. This narrows the margin before heat gain exceeds heat loss and can no longer be offset^12^. A central marker for this heat gain is body core temperature, which typically remains tightly regulated for tens of minutes before responding to increases in microenvironmental temperature. Therefore, when heat stress becomes uncompensable, body core temperature rises progressively^13^. In such extreme heat stress conditions, decision-making in athletes, medical personnel, and firefighters may be impaired leading to risky and impulsive behaviour with far-reaching consequences^3,4^.

Research on core affect and decision-making is limited in heat stress. Laboratory studies inducing affect via media or emotional experiences indicated that positive affect increased risk-taking behaviour, while negative affect reduced it, although some findings reported inverse effects^14^. Core affect can be described as non-reflective feeling that is consciously available if one attends to it^15^. Vitality and fatigue commonly appear to diverge and represent opposite poles in the circumplex model of affect^16–19^. However, the unipolar nature of the scales allows for the expression of mixed feelings when both are experienced with high intensity^15,20^. Typically, vitality and fatigue are assessed via scales or questionnaires^21–23^. The core affective state of subjective fatigue is sensitive to changes in heat stress during passive heat exposure, physical activity, and firefighting^24–26^. Consistent with this, a body core temperature of 38.1°C has been associated with significantly increased fatigue and reduced vigour, with vigour closely related to vitality (described as “psychological energy”)^5^. Vitality can be characterised as a subjective, positive, dynamic, and adaptive state shaped by both physical and psychological energy^27^. It represents an inner personal resource that can be influenced by external conditions, physical factors, and psychological dispositions, all of which are affected by heat stress^5,13,23,27^. Despite its relevance, encompassing drive, energy, and spirit, vitality has been underexplored within the domain of heat research, particularly in its interplay with fatigue^21^.

Impulsivity can impact decision-making processes on multiple levels and is characterised by a tendency to respond quickly without careful planning, reduced sensitivity to the potential negative consequences of actions, and a disregard for long-term outcomes^28^. Evenden^29^ identified three key stages: the preparation stage, the action execution stage, and the outcome phase. During the preparation phase, individuals gather and reflect on available information before making a decision, described as reflection impulsivity by Kagan^30^, which can be assessed using the Beads Task (BT)^31^. Gaoua et al.^3^ found that passive heat stress led to faster responses and less accurate decision-making, indicating heightened impulsivity. However, the specific effect of heat stress on reflection impulsivity needs further investigation.

Impulsivity is often linked to an increased tendency to engage in risk-taking behaviour. Risk-taking behaviour encompasses actions or inactions that involve potential risks^32,33^. Specific impulsive processes are likely to contribute to risky decision-making, although the empirical overlap between impulsivity traits and risk taking measured with the Balloon Analogue Risk Task (BART) is relatively small^34–36^. Chang et al.^4^ reported increased risk-taking behaviour following one hour of heat exposure at 50 °C and 20% relative humidity, suggesting impaired decision-making. However, the lack of physiological and subjective indicators limits the insights into the underlying mechanisms.

Body core temperature is often used as a physiological marker of heat storage^37^. Cognitive performance appears to follow an inverted U-shaped relationship with body core temperature, with impairments emerging around 38.5 °C during complex tasks^38,39^. The maximal adaptability model suggests that heat stress becomes critical when compensatory mechanisms are exceeded, emphasizing the dynamic state of body core temperature rise alongside absolute values^40^. Additionally, exercise duration, repetition, or time spent at elevated body core temperatures influence cognition^41^.

Affect, decision-making, and physiology have often been analysed in isolation; a more holistic approach is needed to understand how heat stress, decision-making and affect are interrelated. Body core temperature serves as a key interoceptive signal, contributing to subjective affect through interoceptive networks^42,43^. Exteroceptive signals, for instance, feeling discomfort when wet clothing sticks to the skin, also contributes to affective appraisal^44^. Gaoua et al.^3^ found that changes in affect rather than changes in body core temperature had a greater influence upon decision-making. Nevertheless, heat stress has been found to separately alter core affective states of subjective fatigue and vitality, as well as impulsivity or risk-taking behaviour^3–5,24,25^. It is therefore important that heat stress severity and duration are standardised and controlled within study designs investigating heat stress and psychophysiological outcomes^39,41^.

The aim of this study was to explore the interrelationships between subjective affective states (vitality, fatigue, thermal sensation [TS], thermal comfort [TC]), and decision-making (risk-taking behaviour and reflection impulsivity) while controlling for physiological parameters (body core temperature, skin temperature, and heart rate) and trait characteristics (impulsivity, sensation seeking and risk-taking).

First, we hypothesised that exposure to heat stress would lead to an increase in fatigue (H1a) and a decrease in vitality (H1b), with these effects differing between passive heat stress and combined heat stress at 38.5 °C, maintained with walking. Variability in these responses may be mediated by individually perceived thermal sensation (TS) and thermal comfort (TC).

Second, we hypothesised that accumulated heat stress would increase risk-taking behaviour and reflection impulsivity. The magnitude of these effects was expected to vary depending on whether heat stress was induced passively or prolonged through maintaining a body core temperature of 38.5 °C via walking. Additionally, the study examined whether changes in core affect, specifically increases in fatigue or decreases in vitality, explain individual differences in risk-taking (H2a) and reflection impulsivity (H2b) under heat stress. For both hypotheses, we checked whether trait levels of impulsivity, sensation-seeking, or risk-taking influence risk-taking behaviour and reflection impulsivity.

## Method

### Participants

The current study included 36 healthy male participants aged 19 to 35 years (*M* = 26.94 years*, SD* = 3.25) with a mean height of 183.05 cm (*SD* = 6.30) and a mean weight of 79.42 kg (*SD* = 9.46; BMI: *M* = 24.25, *SD* = 2.34). All participants were regularly physically active (intensive minutes per week: 61.00, *SD* = 40.24; moderate minutes per week: 62.77, *SD* = 30.15). Exclusion criteria included heat acclimatisation or recent exposure (within the last 30 days) to warmer climates for more than five consecutive days. Participants were also excluded if they were receiving psychotherapeutic treatment.

A repeated-measures, between-groups design was employed, comprising one control group (CG) and two experimental groups: a passive heat stress group (passive group) and a combined heat stress group (combined group). The combined group received passive heat stress followed by two bouts of treadmill walking. The first 24 participants were randomly assigned to one of the three groups (eight per group). For the randomised subset, allocation was concealed using centralised computer assignment. The remaining twelve were matched based on their baseline performance in the BART and BT tasks to ensure comparable cognitive profiles across conditions.

Because males and females exhibit differences in risk-taking, impulsive behaviours, and thermoregulatory responses to heat stress^45–47^, and only 9.5% of firefighters in Austria are female^48^, a homogeneous sample of males was selected.

Ethical approval was obtained from the Ethics Committee of the Paris Lodron University Salzburg (GZ 16/2023). The study adhered to local regulations and institutional guidelines. All participants provided written informed consent. The trial was registered with the German Clinical Trial Register (DRKS—Deutsches Register Klinischer Studien) (DRKS00039790; 27/03/2026).

### Procedure

The study was conducted over the summer season, from May to September. The measurements were split over two days: on the first measurement day, participants were familiarised with study design and the two behavioural tasks (BART, BT) by practicing each test once to ensure comprehension and minimize learning effects. Then participants were assigned to one of three groups: either control group (CG), passive heat stress group or combined heat stress group. In the passive group, heat stress was induced by sauna exposure followed by a sitting phase in protective clothing, withstanding the a priori accumulated heat stress. The combined group completed the sauna exposure as well but performed two additional subsequent walks (Sauna plus two walks à 20min) on a treadmill equipped with protective clothing (Saturn, HP COSMOS, Germany).

On the second day, the experiment started between 8 a.m. and 10 a.m., based on the individual sleep–wake routine of the participants and they were advised to eat 3 h before the experiment, with respect to circadian rhythmicity^49^. Upon arrival, participants’ height and weight were measured (seca 899, seca gmbh & co.kg., Germany) and the hydration status was checked (urine specific gravity < 1.020; PAL-10S handheld refractometer, ATAGO Co Ltd, Japan). Six participants were identified with to high urine specific gravity (*M* = 1.025, *SD* = 0.004) and drank 0.5 L of water before entering the sauna. During the experiment, drinking water was provided ad libitum. The drinking water temperature was heated and controlled via thermometer (Checktemp Electronic Thermometer, Hanna Instruments, USA) to match the current body core temperature to minimise artefacts in the ingested temperature pill. Mean sweat loss was 1.67 kg (*SD* = 0.60) in the combined group and 1.15 kg (*SD* = 0.32) in the sauna group. After correction for ad libitum water intake, the combined and sauna groups lost, on average, 1.31% (*SD* = 0.68) and 0.97% (*SD* = 0.47) of body mass and did not exceed a critical water deficit of 2%^50^. Then, skin temperature sensors (DS1922L-F5 iButton, Maxim Integrated, USA) and an HR chest strap (H10, Polar Electro, Finland) were attached, and a light vest with temperature and humidity sensors (Wireless Climate Network 2, Hochschule Kaiserslautern, Germany) was provided.

Afterwards, they completed the questionnaires and the behavioural tasks BART and BT prior to the heat exposure (T1). To achieve the same time interval between the measurements for the behavioural tasks, the passive group watched a documentary for 20 min. Before entering the sauna, participants of both heat stress groups walked for 10 min at 4 km*h^−1^ to promote peripheral vasodilation and heat production without causing fatigue, to standardise the thermal state^51^. Then they were dressed in firefighter protective clothing (jacket, trouser, gloves, seal hood, TEXPORT GmbH, Austria; mask, MSA Europe GmbH, Switzerland) and sat directly in the sauna for 30 min (passive group: *M* = 60.97 °C, *SD* = 1.00, *M* = 49.04% humidity, *SD* = 8.21; combined group: *M* = 60.02 °C, *SD* = 1.35, *M* = 45.27% humidity, *SD* = 9.08). When entering the sauna, protective clothing initially reduces radiant and convective heat transfer, reducing the heat exposure compared to wearing no protective clothing. However, protective clothing also traps metabolic heat, causing core temperature and heat strain to rise more rapidly thereafter. The humidity was controlled via sauna infusions every five minutes. Two minutes before leaving the sauna they were guided through some muscle activations, to prevent initial orthostatic hypotension^52^.

Directly after leaving the sauna, the passive group completed the behavioural tasks (measurement 2: T2). Subsequently, they continued watching the documentary for 20 min, followed by the third behavioural tasks measurement (T3). The combined group was fitted with a helmet and the air tank of the breathing apparatus (MSA Europe GmbH, Switzerland), but without being connected to the air of the tank.

**Fig. 1:**
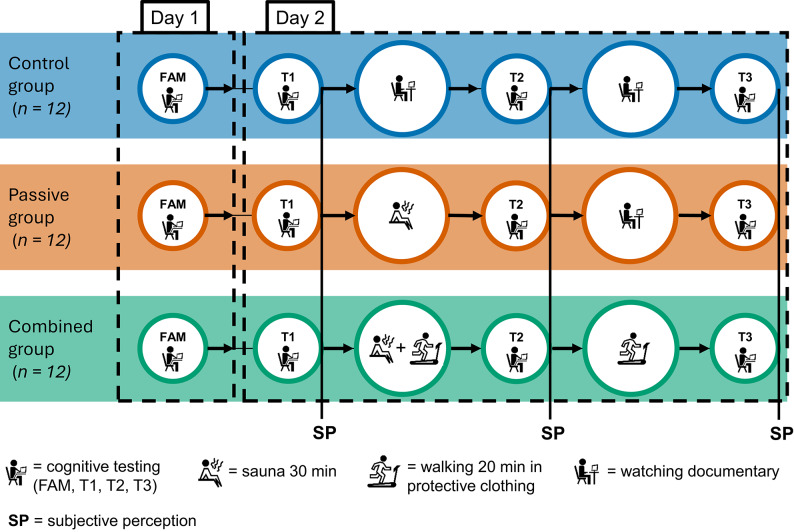
Protocol of the experiment. Timeline of the experiment organised based on content coherence (FAM, familiarisation on day one; T1, pre-intervention; T2, during intervention; T3, post-intervention). Groups are divided into control group (CG), passive heat stress (PHS), and combined heat stress (CHS). Subjective perception (SP) was elicited post behavioural tasks and during sauna, treadmill walking, and documentary every five minutes.

Then, they walked on the treadmill for 20 min to reach a body core temperature of 38.5 °C at the end of the first walking episode. The speed and incline (max. 6 km*h^−1^ at 3%) were adjusted according to this goal^53^. After completing the treadmill walking, they also did the behavioural tasks for the second time on the experimental day (T2). They did not perform the tasks right after the sauna, to avoid a drop in body core temperature. Instead, the passive group accounted for changes directly after the sauna. Then the second 20 min treadmill walk was executed keeping the body core temperature constant at 38.5 °C, followed by the two final behavioural tasks and questionnaires (T3). The CG watched a documentary between the three measurements (T1, T2 and T3), ensuring the same time difference between them in all groups. The evaluation of subjective perception during the behavioural tasks was collected immediately after participants completed both tasks. The subjective measurements for the behavioural tasks were assessed directly post execution. During sauna, treadmill walking, and documentary watching, subjective perception was elicited at five-minute intervals. Immediately after each heat stress intervention, participants remained on site for approximately 30 min. A brief safety check was conducted 24–48 h later via email or phone (Fig. 1).

## Materials

### Questionnaires and scales

Thermal sensation (TS) is a measure of subjective perception, capturing whether an individual feels hot, cold, or neutral. Based on this perception, individuals reflect the physiological and psychological satisfaction with their thermal environment, a concept referred to as thermal comfort (TC)^54^. In a series of studies, TC and TS scales were validated for non-uniform, transient conditions (e.g., experiencing a cold stimulus as comfortable after sauna exposure)^55^. The scale was used as a 21-point version from − 10 to + 10. The combination of TS and TC provides a comprehensive subjective measure for capturing dynamic changes in thermal environments^56^. Over the course of the experiment, the participants were expected to experience transient and/or stable temperature conditions, starting with rising body temperatures in the sauna, stabilising during walking in the combined group, or slightly decreasing temperatures in the passive group after sauna exposure.

Fatigue as Rating-of-fatigue (ROF) and subjective vitality were assessed using a 11-point scale^21,22^. Both are 1-item scales and therefore suitable to measure moment-to-moment changes during the heat stress exposures. The ROF ranges from 0 = not fatigued at all to 10 = total fatigue and exhaustion^22^. Subjective vitality in the present was assessed by the SVS-GM1 (“I feel vital, full of drive and spirited”) from 0 = not true at all to 10 = totally true and as well by the SVS-BM3 pre and post experiment via electronic questionnaire^21,23^.

Affective state was measured using the Positive and Negative Affect Schedule (PANAS)^57,58^. Each item describes a positive or negative affective state (e.g., enthusiastic, inspired, upset, scared). Participantst rated from 1 = not at all to 5 = extremely how much the item correlates with their affective state in the present moment^57^. The PANAS was measured pre- and post the intervention.

Impulsiveness traits were self-reported with the Short-Urgency-Premeditation-Perseverance-Sensation Seeking-Positive Urgency (SUPPS-P) scale^59,60^. The questionnaire was originally designed to quantify four dimensions of impulsivity: negative urgency (tendency to behave impulsively while experiencing intense negative emotions or stress), lack of premeditation (tendency to act without thinking of the consequences), lack of perseverance (inability to remain focused on tasks), and sensation seeking (tendency to seek out novel and thrilling experiences)^59^. Later, Cyders et al.^61^ added the complementary positive urgency dimension (tendency to behave impulsive while experiencing intense positive emotions or excitement). The SUPPS-P consists of 20 items, and each is rated on a 4-point scale from 1 = agree strongly to 4 = disagree strongly. It is reliable and valid compared to the elaborate UPPS-P version. The minimal losses in accuracy are balanced by significant time savings^60^. The traits of risk-taking scale, impulsivity (SUPPS-P) and sensation seeking (SUPPS-P) were assessed prior to the experimental intervention^60^. The trait measures were subsequently incorporated into the statistical mixed modelling analyses.

To assess general willingness to take risk at the trait level a one-item scale was used. The participants were asked “How do you see yourself—how willing are you in general to take risks?”, rating the item from 1 = not at all willing to take risks to 7 = very willing to take risks^62^.

### Balloon Analogue Risk Task (BART)

BART is a computerised task displaying a balloon on a screen^63^. Pressing the ‘right arrow’ key inflates the balloon with each keystroke and adds five points to the participant’s account. At some point, the balloon may burst; if it does, the points from the current trial are lost. The participants can voluntarily inflate the balloon until one decides to collect the points. The goal is to collect as many points as possible. The goal variable for the analysis was the adjusted average pump score (AAP). Typically, the BART consists of 30 trials, with a maximum of 128 pumps possible before the balloon bursts, making 64 pumps per trial the theoretical optimal number. Hence, the probability of explosion increases with every pump until it reaches 1. The participants received no information about the actual possible pumps or breaking points^63^.

Due to three tests within two hours, it was decided to halve the theoretical number of pumps to 64 and utilise just 20 trials, to minimise learning or habituation effects and repetitiveness for the participants^64–66^. A major issue of the BART is the systematic impact of the first trials, to address this issue the first three trials were standardised (trial 1: 37, trial 2: 27, trial 3: 32)^65,67,68^. The task was programmed using E-Prime (Psychology Software Tools, v3.0, https://pstnet.com/products/e-prime/). The BART has been shown to correlate with trait measures of impulsivity, can differentiate groups that show risky behaviour like smoking or extreme sports and to change in response to acute stressors^34,63,69–71^.

### Beads task (BT)

The task was originally designed by Phillips and Edwards^72^ to measure the probability estimates of the participants and how prior gathered information and diagnostic impacts the estimations. Huq et al.^31^ adapted the BT to investigate delusions in schizophrenic patients. In the present study, the task was designed with E-Prime (Psychology Software Tools, v3.0, https://pstnet.com/products/e-prime/). The task starts with the presentation of two jars with the same fixed ratio (e.g., 60:40). Now, one bead is drawn at a time out of one jar and presented to the participant. The participant can respond ‘yes’, I want to gather more information (right arrow on the keyboard), or ‘no’ I have decided to choose a jar (by pressing ‘1’ or ‘2’ on the keyboard). The amount of gathered information is expressed in draws-to-decision (DTD). The sequence of beads presented was for all subjects the same, to ensure the same information basis. Research has shown that participants draw significant more beads in the 60:40 conditions compared to lower beads ratios and in some cases an 80:20 ratio did not reveal any significant differences compared to a 60:40 ratio^35,73^. Another reason the 60:40 ratio was chosen is to make the task more ambiguous, consequently more prone to decisions in real life and more complex in nature as complex tasks are expected to be altered at a body core temperature of 38.5 °C^38^. Following the recommendations of McLean et al.^74^ it was decided to pre-determine the sequences. The sequence of beads has been shown to influence DTD, making it appropriate to standardise the sequence for all participants^74,75^. In total, the participants could draw a maximum of 20 beads per sequence. Five different sequences were presented four times to the participants, resulting in total 20 trials. Two practice sequences were administered at the beginning^74^. To create an incentive, each sequence started with an initial balance of 200 points, with 10 points deducted for every draw^72,76^. If the chosen jar is correct, the participant received the remaining points. If the jar was chosen incorrect no points were made. The goal was to accumulate as many points as possible in total^76^. Total points and points collected in the recent sequence were presented to the participant.

### Temperature and humidity

Body core temperature was measured every 30 s using an ingestible telemetric temperature pill (eCelsius Performance capsule, BodyCap, France), which transmitted the data via radio frequency (433–434 MHz). Participants in the passive or combined group received the ingestible pill with the instruction to ingest it 2–3 h prior to the experiment on the second day. During rest and exercise, it has shown good validity and reliability compared to rectal temperature, making it suitable for portable lab and field use^77,78^.

Skin temperature was measured every 30 s using wireless iButtons (Maxim Integrated, USA), to approximate the thermal gradient from body core to skin^12^. The iButtons were attached to disinfected areas of the skin via tape (Leukoplast waterproof, Beiersdorf AG, Germany). Skin temperature measurement with iButtons is a reliable, valid, and cost-efficient method to measure multiple skin sites^79^. The measurement sites were chosen according to the recommendations of Taylor et al.^80^ relative to the long-term research object to test the cooling effect of an air-cooling jacket. Hence, according to ISO standards (9886:2004) five of the nine sensors were attached to the anticipated cooling area of chest, abdomen, scapula, lower back and shoulder, while the remaining were placed on forehead, upper arm, calf and upper thigh. To assess the thermal climate in the firefighter jacket, it was chosen to integrate nine wireless Bluetooth humidity and temperature sensors in a light vest integrated into custom textile pockets at a sampling rate of 1 Hz^81^. One WCN2 sensor measured air humidity in the sauna, and the temperature was assessed via digital thermometer (Checktemp Electronic Thermometer, Hanna Instruments, USA).

### Statistics

#### Linear mixed models

Given the repeated-measures design and the given hierarchical data structure,we used a mixed-model approach with restricted maximum-likelihood estimation. All linear mixed models (LMM) were set up in R (v4.2.2, https://www.r-project.org/) with *lme4* (v1.1–3.5, https://cran.r-project.org/package=lme4) and checked for linearity, random distribution of residuals and homoscedasticity^82,83^. No data imputation was performed. Each statistical model included a random intercept for participants to account for the repeated-measures nature of the subjective perception and the behavioural tasks. Vitality, fatigue, TS and TC were z-standardised to address multicollinearity resulting in acceptable variance inflation factors (VIF) of < 5 in all models^84^.

LMM was used to model the outcome variables fatigue and vitality, with the assumption of normally distributed data. Given that both BT and BART count their primary outcome variables (DTD, AAP), the distribution of these was modelled accordingly. Since average counts over 20 trials are typically non-integer values, a log-link transformation was employed instead of a Poisson. The two final LMM examined the effects of the fixed factors, group and time, either on reflection impulsivity (DTD) or risk-taking behaviour (AAP). Participants were included using random effects with a random intercept.

According to the hypotheses, we included in the model the interaction for time and group to account for heat stress, also both thermal perceptions (TS and TC) and vitality and/or fatigue. Also, trait characteristics were considered including SUPPS-P, sensation seeking and willingness to take risk when modelling risk-taking behaviour and reflection impulsivity. For TS, TC or vitality/fatigue we included a separate interaction for respectively time and group. Then a stepwise comparison was used, to check whether a triple interaction of each affective state with time and group would improve the model fit. If so, the interaction was included in the model, which was evaluated via Akaike information criterion (AIC), Bayesian information criterion (BIC), alongside an analysis of variance (ANOVA) to check if the changes in log-likelihood ratio were significant. This approach was chosen to prevent an unnecessarily over-complex model, considering the sample size. Methodological and content-related considerations were also taken into account when selecting the final ‘best-fitting’ model. All four final models are reported in the supplementary material as well as detailed stepwise comparisons, random effects, *r*^2^, intraclass correlations (ICC) and model equations.

## Results

### Descriptive statistics

**Fig. 2:**
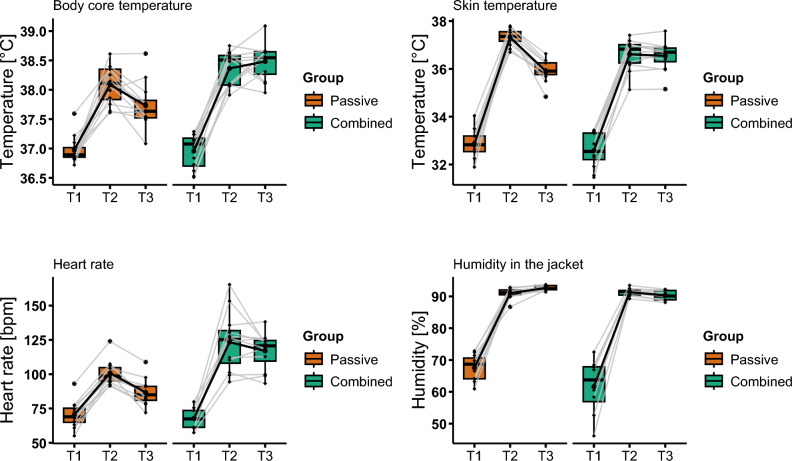
Physiological measurements. Graphics **a**–**d** show boxplots for body core temperature, skin temperature, heart rate and humidity in the jacket. Respectively, the difference between passive and combined group is illustrated, as well as the time (T1, pre intervention; T2, during intervention; T3, post intervention). The grey lines represent individual participants development over time. The bold black lines represent the mean of the groups over time. It must be acknowledged that three data points at T1 are missing for heart rate and four at T1, three at T2 and three at T3 for relativehumidity in the jacket.

The physiological data reported in Fig. 2, suggests that the experimental heat stress manipulation was successful. The targeted temperature criterion was met, reaching a mean body core temperature of 38.4 °C (*SD* = 0.27) at T2 and 38.5 °C (*SD* = 0.40) at T3 in the combined group. In the passive group, the mean body core temperature was 38.1 °C (SD = 0.32) at T2 and 37.7 °C (SD = 0.38) at T3 (Table 1).

**Table 1:** Participants characteristics.

Variable	Control group		Passive group		Combined group	
	*M*	*SD*	*M*	*SD*	*M*	*SD*
Age [years]	26.42	± 2.94	26.50	± 3.32	27.92	± 3.53
Height [cm]	181.79	± 5.29	178.06	± 6.66	183.29	± 6.19
Weight [kg]	82.33	± 9.23	77.43	± 9.6	79.46	± 10.40
Intense phys. activity per week [min]	53.42	± 37.37	59.58	± 42.29	70.00	± 42.59
Moderate phys. activity per week [min]	52.5	± 31.94	79.83	± 33.50	65.00	± 23.65
SUPPS-P [20 items; 1–4]	2.20	± 0.88	2.12	± 0.88	2.04	± 0.89
Sensation seeking [4 Items; 1–4]	2.33	± 0.95	3.23	± 0.77	3.15	± 0.85
Risk-taking scale [Single Item 1–7]	4.66	± 1.37	5.08	± 1.00	4.92	± 1.08
Positive Affect [10 items 1–5]	2.87	± 1.15	3.18	± 1.08	3.47	± 1.42
Negative Affect [10 items 1–5]	1.34	± 0.98	1.22	± 0.45	1.30	± 0.64

The descriptive results of demographic characteristics, physical activity, PANAS and SUPPS-P are summarised. All data were collected prior to the interventions.

**Fig. 3:**
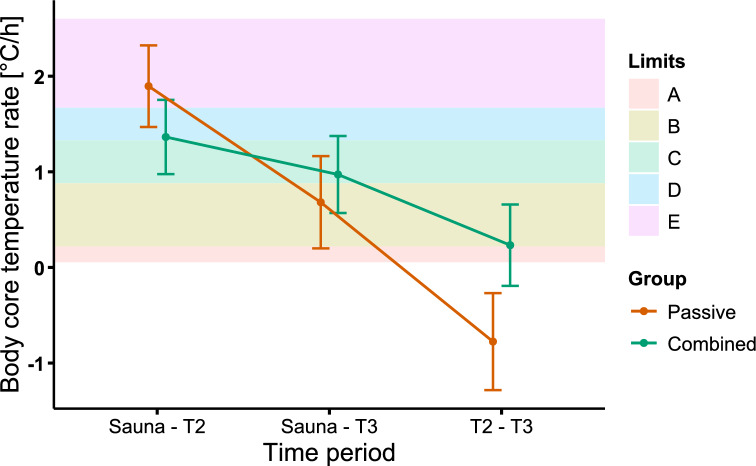
Change rate in body core temperature during selected time periods. The difference between passive and combined group is illustrated. The coloured lines represent the groups change rate and standard deviation during selected time periods. The average body core temperature of the first 5 min in the sauna and the average body core temperature during T2 (during intervention) and T3 (post intervention) was used to calculate the change rate (°C/h), with respect to the mean time difference. The coloured areas represent the limits proposed by Hancock and Vasmatzidis^40^ for different cognitive task types (A: Vigilance, 0.055 °C/h; B: Dual-task, 0.22 °C/h; C: Tracking, 0.88 °C/h; D: Simple mental, 1.33 °C/h; E: Physiological tolerance, 1.67 °C/h).

The rise in body core temperature from the first five minutes of the sauna to either T2 or T3, and from T2 to T3, was calculated and divided by the mean time difference between measurements. It has been suggested that the rate at which body core temperature rises per hour is a central physiological measure for estimating cognitive performance declines in heat stress^40^. The mean time difference from sauna to T2 or T3 and T2 to T3 was used to calculate the degree Celsius per hour rate (Fig. 3).

### Effects of heat stress on vitality and fatigue

Evaluating fatigue hypothesis 1a, the ‘basic’ model included the interaction of time and group, the subjective measures of vitality, TC and TS, with all three separately interacting with time and group. In this way, the effect over time and between groups was tested separately, compared to the control group at T1. Stepwise comparison revealed that the model fit did not significantly improve when adding the triple interaction of time x group and vitality (χ^2^(4) = 5.17, p = 0.27), TC (χ^2^(4) = 3.01, p = 0.55), TS (χ^2^(4) = 4.53, p = 0.34) or of all three (χ^2^(4) = 16.69, p = 0.16). Hence, the ‘basic’ model with separate time and group interactions for each subjective measure was chosen.

Compared to T1, fatigue decreased to T3 in the control group (β = − 0.50, *p* = 0.020, 95% CI [− 0.92, − 0.08]). In the passive group at T2, fatigue was increased by 0.80 (*p* = 0.034, 95% CI [− 0.06, 1.54]) and by 0.95 at T3 (*p* = 0.002, 95% CI [0.36, 1.55]) compared to the control group at T1. In the combined group significant increases were found to T3 (β = 0.82, *p* = 0.037, 95% CI [0.05, 1.59]). Independent of group and time, decreases in vitality led to significant increases in fatigue (β = − 0.98, *p* < 0.001, 95% CI [− 1.31, − 0.65]), revealing that a 1-unit increase in vitality is associated with a 0.98-unit decrease in fatigue. At T3, this strong relation is reduced by 0.42 (*p* = 0.006, 95% CI [0.13, 0.71]), meaning that a 1-unit increase in vitality is associated with a 0.56-unit decrease. Finally, TS had a significant increasing effect on fatigue at T2 (β = 0.33, *p* = 0.050, 95% CI [0.00, 0.65]). The intraclass correlation (ICC) of 0.48 showed that substantial variance can be explained by between-subject variance. The model explained 61.4% of the variance by fixed effects (${r}_{marginal}^{2}$) and 80.1% by fixed and random effects (${r}_{conditional}^{2}$).

For the model evaluating H1b, with vitality as outcome variable, the same approach was chosen as in the fatigue model. The stepwise comparison indicated significant improvement for the triple interaction with TC (χ^2^(4) = 15.27, p = 0.004, AIC = 206.31, BIC = 286.77), the triple interaction with TS showed a worse model fit further in terms of higher AIC and BIC (AIC = 219.55, BIC = 300.01). Including triple interactions for all subjective measures did not improve the fit (χ^2^(8) = 6.82, p = 0.56). So, it was decided to take the model with the triple interaction for TC, indicating the best overall fit.

The vitality model showed no significant effects over time and group in vitality, but the relations to the subjective measures did. In the control group increases in fatigue were associated with lower vitality (β = − 0.22, *p* = 0.022, 95% CI [− 0.41 − 0.03]), being significantly more influential at T3 (β = − 0.22, *p* = 0.022, 95% CI [− 0.41 − 0.03]). In the passive group this association was generally significantly stronger (β = − 0.39, *p* = 0.029, 95% CI [− 0.73, − 0.04]). As suggested in the stepwise comparison, TC had a significant role in predicting vitality. TC had a significant positive influence on vitality in the control group (β = 0.59, *p* < 0.001, 95% CI [0.28, 0.91]). In both heat stress groups this relation was significantly weaker with a net effect of − 0.11 in the passive group (β = − 0.70, *p* < 0.001, 95% CI [− 1.12, − 0.29]) and of 0.12 in the combined group (β = − 0.47, *p* = 0.020, 95% CI [− 0.86, − 0.07]). Further, in the control group the influence of TC on vitality was significantly weaker at T3 (β = − 0.65, *p* = 0.004, 95% CI [− 1.08, − 0.21]).

**Fig. 4:**
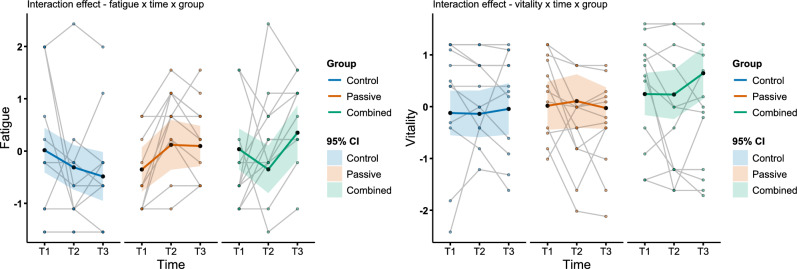
Subjective perception of fatigue and vitality. Graphics **a** and **b** show the fixed effects as the coloured bold line for fatigue and vitality, with 95% confidence intervals (CI) represented in the shaded areas. Respectively, the difference between control, passive and combined group is illustrated, as well as the time (T1, pre intervention; T2, during intervention; T3, post intervention). The points represent individuals of the groups over time.

Examining the triple interaction revealed that the effect of TC at T2 on vitality was significantly more positive in the passive group (β = 0.54, *p* = 0.048, 95% CI [0.00, 1.08]) and the combined group (β = 0.71, *p* = 0.013, 95% CI [0.16, 1.26]). Especially at T3 the passive (β = − 0.97, *p* = 0.002, 95% CI [0.37, 1.56]) and the combined group (β = 1.12, *p* < 0.001, 95% CI [0.51, 1.73]) had a significantly more positive impact of TC on vitality. The fixed effects explained 48% of variance, and together with the random effect 88% of the variance. The ICC of 0.77 showed that great parts of the variance are due to between-subject differences (Fig. 4).

### Effects of heat stress on risk-taking behaviour and reflection impulsivity

Evaluating H2a, the ‘basic’ linear mixed model with risk-taking behaviour as outcome variable included as fixed effects the group and time interaction, all four subjective measures (fatigue, vitality, TC and TS), as well as three traits (SUPPS-P, sensation seeking and willingness to take risk). Similar to the fatigue and vitality model, the triple interaction for each subjective measure was introduced via stepwise comparison and was included in the model when the interaction led to a significant model improvement.

In the first step, fatigue did not improve the model (χ^2^(4) = 4.63, p = 0.33), but vitality did (χ^2^(4) = 10.10, p = 0.038, AIC = − 61.11, BIC = 40.81) and was included in the model. Further comparing to the TC only interaction model led to no improvement (AIC = − 57.44, BIC = 44.49). The triple interaction with TS did improve the model compared to the vitality interaction (AIC = − 65.45, BIC = 36.47). Then, adding interactions for both vitality and TS, made the model worse compared to the TS model (χ^2^(4) = 2.19, p = 0.70), so it was chosen to favour the TS model. Finally, adding triple interactions for all subjective measures did not further improve the model (χ^2^(12) = 9.69, p = 0.64).

The ‘best’ fitting TS model revealed that the interaction between group and time was significant, showing an increase in adjusted average pumps (AAP) for the combined group at T3 (β = 0.17, *p* = 0.023, 95% CI [0.02, 0.31]). Hence, the combined group increased AAP to T3 by 18.5% compared to the control group. Also, the predictive power of fatigue and vitality changed at T3. Increases in fatigue (β = − 0.10, *p* = 0.013, 95% CI [− 0.17, − 0.02]) or vitality (β = − 0.09, *p* = 0.013, 95% CI [− 0.16, − 0.02]) decreased AAP by 9.5% or 8.7%. Further, the combined group showed a negative relation of AAP and TS (β = − 0.11, *p* = 0.022, 95% CI [− 0.21, − 0.02]) compared to the control group. In the passive group the relation of AAP and TS was significantly more negative compared to T1 in the control group (β = − 0.27 *p* = 0.001, 95% CI [− 0.42, − 0.11]). Thus, an increase of one unit in TS led to 24% lower AAP. The fixed effects explained 13.3% of the variance (${r}_{marginal}^{2}$) with fixed and random effect explaining 93.4% (${r}_{conditional}^{2}$), which is in line with the high ICC of 0.92 attributing the variance to a great part on between-subject differences.

**Fig. 5:**
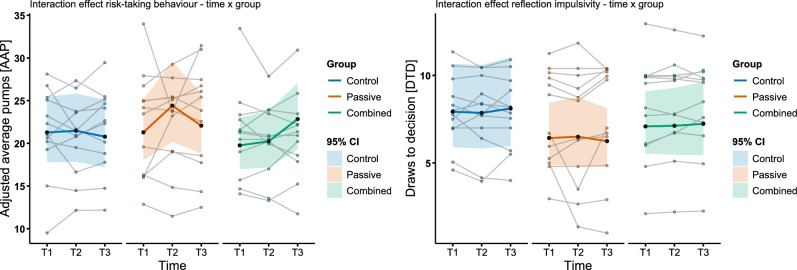
Decision-making tasks. The Balloon Analogue Risk Task (BART) measured risk-taking behaviour, and the Beads Task (BT) measured reflection impulsivity. Graphics **a** and **b** show the fixed effects as the coloured bold line for risk-taking and reflection impulsivity, with 95% confidence intervals (CI) represented in the shaded areas. Respectively, the difference between control, passive and combined group is illustrated, as well as the time (T1, pre intervention; T2, during intervention; T3, post intervention). The points represent individuals of the groups over time.

The model comparison for reflection impulsivity testing H2b, showed that including the triple interaction for fatigue (*χ*2(4) = 5.11, *p* = 0.28), vitality (*χ*2(4) = 3.20, *p* = 0.52), TC (*χ*2(4) = 3.88, *p* = 0.42), TS (*χ*2(4) = 3.31, *p* = 0.51) or all subjective measures (*χ*2(16) = 13.97, *p* = 0.60) did not improve the model. Accordingly, the ‘basic’ model with separate interaction for group and time was chosen as best fitting model. In the passive group, TC negatively correlated with draws to decision (DTD) (β = − 0.21 *p* = 0.012, 95% CI [− 0.37, − 0.11]), compared to the control group. Increase of one-unit in TC led to 19% less DTD. Fixed effects were explaining 18.3% of the variance (${r}_{marginal}^{2}$) with fixed and random explaining 90.7% $({r}_{conditional}^{2}$). The high ICC of 0.89 ascribes significant variance to between-subject differences (Fig. 5).

## Discussion

The overarching objective of the present study was to examine the interrelationships between affect (vitality and fatigue) and decision-making (risk-taking behaviour and reflection impulsivity) during two types of heat stress. Passive and combined heat stress were used as primary stimuli to disturb physiological homeostatic states. Internal afferent visceral signals of hyperthermia were operationalised as body core and skin temperature, representing the bodily interoceptive state. The perception of hyperthermia signals was assessed using thermal sensation (TS) and thermal comfort (TC) integrating internal and external signals. While previous research has shown that heat stressors can affect subjective perception, behavioural risk-taking, and impulsivity separately, an explicit link between types of heat stress, affect, and decision-making behaviour requires empirical evidence^3,4^. This study aimed to address this gap, contributing to a better understanding of subjective perception and human behaviour in the context of heat.

According to H1a, fatigue increased over time in both heat stress groups, in comparison to the control group. This finding aligns with previous research demonstrating sensitivity of fatigue to heat stress^24–26^. Vitality scores were associated with lower fatigue, independent of group and time, which reflects a protective effect of vitality on fatigue. During the experiment, this protective mechanism was significantly weaker, but still present. Also, if the thermal environment was perceived as hotter, feelings of fatigue were stronger during the experiment.

Contrary to hypothesis 1b, vitality did not significantly decrease in either heat stress group. Perceiving a heat stressor as more thermally comfortable was associated with feeling more vital. This suggests that if one manages to improve individual TC, they feel vital and energised, independent of the type of heat stress. In general, increases in fatigue were linked to lower vitality with an increasing impact over time. The negative effect of fatigue on vitality was particularly strong in the passive group. This was not the case in the combined group, suggesting that walking may have a stimulating or distracting effect, making one feel vital even when fatigued and thus making the experience of mixed feelings more plausible.

In summary, feeling more vital has shown to reduce fatigue, while higher fatigue predicted lower vitality. This bidirectionality is consistent with other experimental observations^5^ and with the circumplex model in which fatigue and vitality represent opposing yet connected facets of affect^18,19^. However, both perceptions responded in different ways to heat stress. Fatigue was being influenced directly and vitality being influenced indirectly via TC. This aligns with previous observations from a momentary assessment study which described that vitality and fatigue share similar variance but exhibiting distinct effects that differentiate the two concepts^16^.

Partly supporting the hypothesis H2a, the magnitude of risk-taking behaviour change seems to vary with the type of heat stress and its severity. Increased risk-taking behaviour was observed when a body core temperature of 38.5 °C was reached and then prolonged using combined heat stressors. However, reaching 38.5 °C without maintaining it did not lead to significant behavioural risk-taking changes. This emphasises that alongside the magnitude of body core temperature the duration of hyperthermic states is relevant. However, after the sauna exposure the passive group indicated a trend toward increased risk-taking behaviour (as indicated by the confidence intervals, without being significant). This might reflect that risk-taking behaviour in the passive group is affected by heat stress, but to a smaller degree than the combined group. Hence, both the duration and magnitude of body core temperature must be acknowledged when assessing heat stress severity and risk-taking behaviour. At 38.5 °C the duration might contribute this difference. This was addressed in a review that emphasised exercise duration as an important factor^41^.

The peak skin temperature and its rate of change were observed immediately post-sauna exposure, which both contribute substantially to cognition in the heat^3,40^. It is suggested that body core temperature rises are central to anticipate the point of physiological collapse and thus avoid fatal heat stroke^85^. Within 30 min of passive heat exposure, body core temperature increased at a rate of 1.90 (*SD* = 0.08) per hour in the passive group, which is above the proposed limit of detrimental effects on simple mental tasks and physiological tolerance (1.33–1.67 °C/h)^40^.

Interestingly, more pronounced TS after the passive sauna exposure led to a more risk averse behaviour, which was already found to be closely related to skin and body core temperature^55^. These findings counteract the trend that passive heat non significantly increased risk-taking behaviour, highlighting the complex interplay of bodily heat stress and subjective perception. This finding extends and refines the research that reported significant increases in risk-taking behaviour under heat stress^4^.

Feeling more vital or fatigued led to more risk averse behaviours, independent of the condition. This effect indicates that the BART was more susceptible to affective changes when completed for the third time in about two hours. The findings demonstrate that subjective perception exerts a significant influence on risk behaviour, which supports the findings previously evidenced in broader, heat-unrelated frameworks^14^. While our experimental approach to manipulate affect through heat stress shares conceptual and methodological similarities with prior studies, differences in behavioural tasks, affective measures, and especially affect manipulation techniques limit direct comparability^14^.

Not supporting hypothesis 2b, there was no significant influence of both heat stressors on reflection impulsivity in comparison to the control group. However, we found that the information gathering process was significantly shortened when individuals felt more thermally comfortable in the passive group. The result may seem counterintuitive if decision-making is more deliberate when feeling thermally comfortable. According to these results, feeling thermally comfortable during heat stress may put individuals in a self-evident attitude, which leads them to act and make decisions faster. Additionally, it should be noted that some people find the experience of heat in the sauna as pleasant, while others do not, leading to TC creating sublevel structures^86^.

The behavioural stability may suggest that the observed patterns were shaped by trait-like characteristics, rather than by state-dependent factors. However, this interpretation conflicts with the present finding that the trait questionnaires did not significantly predict DTD. Alternatively, the consistency may indicate a memory effect as participants rely on a previously-learned behavioural pattern. This raises questions about short-term reliability and validity of BT performance in repeated measures designs^74,87^.

Several factors and pathways have been drawn on how heat stress influences risk-taking behaviour. The maximal adaptability model by Hancock and Vasmatzidis^40^ systematically describes the mechanisms of heat stress affecting cognition. Referring to the maximal adaptability model, individuals in the passive group were still able to psychologically adapt and regulate the sauna heat stressor to a certain degree. One would localise this observation at the outer psychological adaptability zone where psychological compensation is still effective, but nevertheless cognitive performance starts to decrease. Additional walking increased again the adaptive capacity, before the prolonged heat stress duration gradually diminished compensatory psychological resources and pushed towards physiological boundaries^40^.

In this study, when heat stress signals changed in intensity or were maintained over time, the judgement of these signals also changed, manifesting itself in altered affective states. Hence, in heat stress interoceptive signalling is shaped not only by core temperature magnitude but also by its duration, rate of change and skin temperature. However, this physiological information alone was not sufficient to statistically explain affective states of fatigue and vitality. On the one hand, they appeared to influence each other, on the other they were driven by thermal perceptions. Arguably, these thermal perceptions emerged from interoceptive and exteroceptive signal appraisal such as external temperature or from heat and moisture building up under protective clothing. This connects to the understanding that affect arises from integrating interoceptive and exteroceptive information^44^.

This study also illustrates that risk-taking behaviour arises from an intertwined network of body temperatures, vitality, fatigue, TS and TC. These findings might be anchored and explained by the somatic marker hypothesis: the arising interoceptive and exteroceptive heat stress information are pre-shaped by the vagus nerve and processed on an emotional level in the prefrontal areas of the brain. This creates an afferent and efferent feedback loop, evaluating the present bodily state and external or anticipated states, which under heat stress may pose a major threat to bodily functioning and cognition in the present or future^85,88,89^. Synthesized through affective states, exteroceptive and interoceptive heat stress signals highly influenced risk-taking behaviour. The current findings extend this framework to heat stress, shedding light on the interdependencies between bodily sensations, affect and risk-taking behaviour.

### Limitations

The sample size of this study may have limited the power to detect small but meaningful effects. Monte Carlo sensitivity analysis suggested that 200 participants (Level 2) across three groups (Level 1) would be required to detect medium cross-level interaction effects (γ01.std = 0.30), to achieve 80% power (α = 0.05), assuming a large intraclass correlation (ICC = 0.50) (Arend & Schäfer, 2019). However, due to resource limits, the final sample size consisted of 36 participants across three groups. With the current sample size, the study was adequately powered to detect medium to large within-person effects (γ10.std = 0.32), large between-person effects (γ01.std = 0.51), and large cross-level interaction effects (γ01.std = 0.68).

The short-term reliability and validity of the BART and BT in acute settings remain unclear, requiring methodological refinement^34,74^.

Previous heat stress studies were predominantly conducted in autumn and winter with non-heat-adapted individuals. In contrast, the present study was conducted between May and September, thus, the participants were likely heat-adapted. This could reduce the impact of heat stress on cognition^90^.

The ingestion of temperate drinking water (relative to body core temperature) might have affected body core temperature measured by the ingested pill. For ethical and safety reasons, hydration had to be ensured. Ingesting the pill more than 3 h before testing might reduce susceptibility to drinking water artifacts^91^. However, no studies have examined the effect of temperate drinking water relative to core temperature on ingestible pills.

The inclusion of only young healthy males limits the generalisability of its findings to other populations. It remains unclear whether the observed effects would extend to females, older adults, or individuals with pre-existing health conditions. Future studies should consider broader participant demographics.

Given that our sample consisted of non-firefighters, we refrained from drawing any conclusions related to firefighting populations. The unique physiological and psychological demands experienced by firefighters may yield different risk or impulsive responses to heat stress and should be investigated in further experiments.

## Conclusion

The current findings highlight the complexity of determining heat stress severity which in turn affects physiological and subjective signals and finally increases risk-taking behaviour during prolonged and quickly rising heat stress. Subjective perception, capturing interoceptive and exteroceptive signals, appears to play a crucial role in altering risk-taking behaviour during heat stress. While it may introduce bias, precise interoceptive and exteroceptive perception might be a valuable tool for individuals to assess the individual severity of heat stress. Understanding these pathways could help us to prepare and develop strategies for individuals in the face of heat stress. In high-stakes contexts such as athletic performance, emergency medical care, or firefighting, where decision-making can have far reaching consequences, it is crucial to identify and support these processes.

## Supplementary Information

Supplementary Information.

## Data Availability

The data for analysis are publicly available at https://osf.io/5n3py/overview?view_only=3de152859ea84538b467617dc89d5c48, including decision-making metrics, physiological measures, and questionnaire responses. Further requests to access the data should be directed to jannic.waelde@plus.ac.at.
