# Supplementary material for: Heat stress impacts affective processes and risk-taking behaviour
Source: Sci Rep. 2026 Apr 9;16:16721. doi: 10.1038/s41598-026-47250-x (PMC13223210; doi:10.1038/s41598-026-47250-x)
Supplement: Supplementary file 1 — Supplementary Information. [file 41598_2026_47250_MOESM1_ESM.pdf]

# Supplementary material

# Heat Stress impacts fatigue, vitality and risk-taking behaviour: A repeated-measures between-groups experiment


## Author information

[1]Wälde, Jannic, jannic.waelde@plus.ac.at, ORCID: https://orcid.org/0009-0001-1220-9923

[1]Amesberger, Günter, guenter.amesberger@plus.ac.at, https://orcid.org/0000-0002-3078-5326

[1]Würth, Sabine, sabine.wuerth@plus.ac.at, https://orcid.org/0000-0003-2736-2004

[1,2]Reichert, Markus, markus.reichert@plus.ac.at, https://orcid.org/0000-0003-3290-0962

[1]Finkenzeller, Thomas, thomas.finkenzeller@plus.ac.at, https://orcid.org/0000-0003-2736-2004

## Institutional affiliation

[1] Department of Sport and Exercise Science, Paris Lodron University Salzburg, Schloßallee 49, 5400 Hallein, Austria

[2] Department of Psychiatry and Psychotherapy, Central Institute of Mental Health, Medical Faculty Mannheim, Heidelberg University, Mannheim, Germany

## Author Note

Correspondence to Jannic Wälde, Paris Lodron University Salzburg, Schloßallee 49, 5400 Hallein, Austria, jannic.waelde@plus.ac.at


## Abbreviations

Testing before heat exposure (T1), during (T2) and post (T3), rate-of-fatigue (ROF), thermal comfort (TC), thermal sensation (TS), Short-Urgency-Premeditation-Perseverance-Sensation Seeking-Positive Urgency (SUPPS-P), Akaike information criterion (AIC), Bayesian information criterion (BIC), likelihood (LL), random effect (RE), fixed effect (FE), likelihood-ratio test (LRT), intraclass correlation (ICC), standard errors (*SE*), standard deviation (*SD*), 95% confidence intervals (CI), t-values (*t*), p-values (*p*), intraclass correlation (ICC) and r-squared ($r^2$ conditional and marginal)

Supplementary Table 1

Rate of Fatigue (ROF) Mixed Model comparison and the model building/selection process

| **Model** | **Nested / simpler Model** | **Random effects** | **Fixed effects** | **Model fit** | | | | | **LRT against nested** | |
|---|---|---|---|---|---|---|---|---|---|---|
| | | | | **AIC** | **BIC** | **LL** | **df** | **deviance** | **X²** | ***p*** |
| Separate interaction | - | Intercept participants | Timepoint x group + vitality + vitality x time + vitality x group + TC + TC x time + TC x group + TS + TS x time + TS x group | 234.35 | 304.09 | -91.18 | - | 182.35 | - | - |
| Separate interaction + triple interaction vitality | Separate interaction | Intercept participants | Timepoint x group + vitality + vitality x time x group + TC + TC x time + TC x group + TS + TS x time + TS x group | 237.18 | 317.65 | -88.59 | 4 | 177.18 | 5.17 | .27 |
| Separate interaction + triple interaction TC | Separate interaction | Intercept participants | Timepoint x group + vitality + vitality x time + vitality x group + TC + TC x time x group + TS + TS x time + TS x group | 239.30 | 319.76 | -89.65 | 4 | 179.30 | 3.05 | .55 |
| Separate interaction + triple interaction TS | Separate interaction | Intercept participants | Timepoint x group + vitality + vitality x time + vitality x group + TC + TC x time + TC x group + TS + TS x time x group | 237.82 | 318.29 | -88.91 | 4 | 177.82 | 4.53 | .34 |
| Triple interaction | Separate interaction | Intercept participants | Timepoint x group + vitality + vitality x time x group + TC + TC x time x group + TS + TS x time x group | 241.66 | 343.58 | -82.83 | 12 | 165.66 | 16.69 | .16 |

| ROF final model: Separate interaction | | | | | |
|---|---|---|---|---|---|
| | Fixed Effects | | | | |
| **Model parameter** | **Estimate/β** | ***SE*** | **95% CI** | ***t*** | ***p*** |
| Intercept | 0.02 | 0.21 | -0.41 – 0.44 | 0.08 | .938 |
| T2 | -0.33 | 0.21 | -0.74 – 0.08 | -1.60 | .114 |
| T3 | -0.50 | 0.21 | -0.92 – -0.08 | -2.37 | **.020** |
| Group PHS | -0.37 | 0.29 | -0.95 – 0.22 | -1.25 | .214 |
| Group CHS | 0.02 | 0.29 | -0.55 – 0.59 | 0.07 | .946 |
| Group PHS X T2 | 0.80 | 0.37 | 0.06 – 1.54 | 2.16 | **.034** |
| Group PHS X T3 | 0.95 | 0.30 | 0.36 – 1.55 | 3.18 | **.002** |
| Group CHS X T2 | -0.06 | 0.34 | -0.74 – 0.62 | -0.17 | .866 |
| Group CHS X T3 | 0.82 | 0.39 | 0.05 – 1.59 | 2.12 | **.037** |
| Vitality | -0.98 | 0.17 | -1.31 – -0.65 | -5.87 | **<.001** |
| Vitality X Group PHS | 0.30 | 0.21 | -0.11 – 0.72 | 1.45 | .151 |
| Vitality X Group CHS | 0.21 | 0.20 | -0.19 – 0.62 | 1.04 | .303 |
| Vitality X T2 | 0.42 | 0.15 | 0.13 – 0.71 | 2.84 | **.006** |
| Vitality X T3 | 0.22 | 0.14 | -0.05 – 0.50 | 1.60 | .114 |
| TC | 0.25 | 0.21 | -0.16 – 0.66 | 1.23 | .224 |
| TC X Group PHS | -0.27 | 0.22 | -0.70 – 0.17 | -1.22 | .225 |
| TC X Group CHS | -0.27 | 0.23 | -0.74 – 0.19 | -1.16 | .248 |
| TC X T2 | -0.23 | 0.18 | -0.60 – 0.14 | -1.25 | .214 |
| TC X T3 | 0.00 | 0.20 | -0.39 – 0.39 | 0.01 | .993 |
| TS | -0.16 | 0.16 | -0.48 – 0.17 | -0.96 | .341 |

| | | | | | |
|---|---|---|---|---|---|
| TS X Group PHS | 0.06 | 0.24 | -0.42 – 0.54 | 0.25 | .802 |
| TS X Group CHS | 0.23 | 0.20 | -0.17 – 0.63 | 1.13 | 0.260 |
| TS X T2 | 0.33 | 0.16 | -0.00 – 0.65 | 1.99 | **0.050** |
| TS X T3 | 0.12 | 0.16 | -0.20 – 0.44 | 0.77 | 0.441 |

| | **Random Effects** | |
|---|---|---|
| | **Variance** | ***SD*** |
| Participant (Intercept) | 0.19 | 0.44 |
| Residual | 0.20 | 0.45 |

| | **Model fit** | |
|---|---|---|
| | **Marginal** | **Conditional** |
| ***r²*** | .614 | .801 |
| **ICC (Participant)** | | .48 |

The response ROF variable $\gamma_i$ is modelled as:

$$\gamma(\text{ROF})_i = \beta_0 + \beta_1 * time_i + \beta_2 * group_i + \beta_3 * (time_i * group_i) + \beta_4 * vitality_i + \beta_5 * (time_i * vitality_i) + \beta_6 * (group_i * vitality_i) + \beta_7 * TC_i + \beta_8 * (time_i * TC_i) + \beta_9 * (group_i * TC_i) + \beta_{10} * TS_i + \beta_{11} * (time_i * TS_i) + \beta_{12} * (group_i * TS_i) + u_{i0} + \epsilon_i$$

- $\gamma_i$: response for the *i*-th observation.
- $\beta$: fixed intercept
- $u_{i0}$: random intercept for the *i*-th participant.
- $\epsilon_i$: residual error term for the *i*-th observation.
- The random effects structure: $u_{i2} \sim N(0, \sigma^2)$

*Note*. All subjective variables (vitality, ROF, TS, TC) were z-standardised prior to the analysis. The β-values represent the standardized fixed effects predicting ROF. Random intercepts were specified for participants. Bootstrapping with 1000 iterations for all values was used. Results reaching the significance threshold of p < .05 were marked in bold.

Supplementary Table 2
Vitality Mixed Model comparison and the model building/selection process

| Model | Nested / simpler Model | Random effects | Fixed effects | Model fit | | | | | LRT against nested | |
|---|---|---|---|---|---|---|---|---|---|---|
| | | | | AIC | BIC | LL | df | deviance | $X^2$ | *p* |
| Separate interaction | - | Intercept participants | Timepoint x group + fatigue + fatigue x time + fatigue x group + TC + TC x time + TC x group + TS + TS x time + TS x group | 213.58 | 283.32 | -80.79 | - | 161.58 | - | - |
| Separate interaction + triple interaction fatigue | Separate interaction | Intercept participants | Timepoint x group + fatigue + fatigue x time x group + TC + TC x time + TC x group + TS + TS x time + TS x group | 218.72 | 299.19 | -79.36 | 4 | 158.72 | 76.08 | .58 |
| Separate interaction + triple interaction TC | Separate interaction | Intercept participants | Timepoint x group + fatigue + fatigue x time + fatigue x group + TC + TC x time x group + TS + TS x time + TS x group | 206.31 | 286.77 | -73.16 | 4 | 146.31 | 15.27 | **.004** |
| Separate interaction + triple interaction TS | Separate interaction + triple interaction TC | Intercept participants | Timepoint x group + fatigue + fatigue x time + fatigue x group + TC + TC x time + TC x group + TS + TS x time x group | 219.55 | 300.01 | -79.77 | 0 | 159.55 | 0 | - |
| Triple interaction | Separate interaction + triple interaction TC | Intercept participants | Timepoint x group + fatigue + fatigue x time x group + TC + TC x time x group + TS + TS x time x group | 215.49 | 317.41 | -69.74 | 8 | 139.49 | 6.82 | .56 |

| **Vitality final model:** Separate interaction + triple interaction TC | | | | | |
|---|---|---|---|---|---|
| | **Fixed Effects** | | | | |
| **Model parameter** | **Estimate/*β*** | ***SE*** | **95% CI** | ***t*** | ***p*** |
| Intercept | -0.12 | 0.22 | -0.55 – 0.31 | -0.57 | .573 |
| T2 | -0.02 | 0.17 | -0.36 – 0.33 | -0.10 | .922 |
| T3 | 0.08 | 0.19 | -0.31 – 0.46 | 0.41 | .687 |
| Group PHS | 0.14 | 0.32 | -0.49 – 0.77 | 0.44 | .662 |
| Group CHS | 0.11 | 0.36 | -0.62 – 0.83 | 0.29 | .770 |
| Group PHS X T2 | -0.12 | 0.28 | -0.67 – 0.43 | -0.45 | .657 |
| Group PHS X T3 | 0.01 | 0.28 | -0.54 – 0.56 | 0.03 | .975 |
| Group CHS X T2 | 0.32 | 0.30 | -0.27 – 0.92 | 1.09 | .280 |
| Group CHS X T3 | 0.11 | 0.36 | -0.62 – 0.83 | 0.29 | .770 |
| ROF | -0.22 | 0.10 | -0.41 – -0.03 | -2.34 | **.022** |
| ROF X Group PHS | -0.39 | 0.17 | -0.73 – -0.04 | -2.23 | **.029** |
| ROF X Group CHS | -0.13 | 0.14 | -0.41 – 0.15 | -0.94 | .348 |
| ROF X T2 | -0.12 | 0.11 | -0.34 – 0.10 | -1.09 | .279 |
| ROF X T3 | -0.32 | 0.11 | -0.54 – -0.10 | -2.94 | **.004** |
| TC | 0.59 | 0.16 | 0.28 – 0.91 | 3.74 | **<.001** |
| TC X Group PHS | -0.70 | 0.21 | -1.12 – -0.29 | -3.36 | **.001** |
| TC X Group CHS | -0.47 | 0.20 | -0.86 – -0.07 | -2.37 | **.020** |
| TC X T2 | -0.35 | 0.20 | -0.74 – 0.05 | -1.74 | .086 |
| TC X T3 | -0.65 | 0.22 | -1.08 – -0.21 | -2.98 | **.004** |
| TC X Group PHS X T2 | 0.54 | 0.27 | 0.00 – 1.08 | 2.01 | **.048** |

| | | | | | |
|---|---|---|---|---|---|
| TC X Group PHS X T3 | 0.97 | 0.30 | 0.37 – 1.56 | 3.22 | **.002** |
| TC X Group CHS X T2 | 0.71 | 0.28 | 0.16 – 1.26 | 2.55 | **.013** |
| TC X Group CHS X T3 | 1.12 | 0.31 | 0.51 – 1.73 | 3.66 | **<.001** |
| TS | -0.01 | 0.14 | -0.29 – 0.27 | -0.06 | .950 |
| TS X Group PHS | 0.03 | 0.22 | -0.42 – 0.47 | 0.12 | .906 |
| TS X Group CHS | 0.15 | 0.18 | -0.21 – 0.51 | 0.84 | .404 |
| TS X T2 | -0.10 | 0.13 | -0.36 – 0.16 | -0.77 | .443 |
| TS X T3 | 0.07 | 0.12 | -0.18 – 0.31 | 0.53 | .595 |

| | **Random Effects** | |
|---|---|---|
| | **Variance** | ***SD*** |
| **Participant (Intercept)** | 0.34 | 0.58 |
| **Residual** | 0.10 | 0.32 |

| | **Model fit** | |
|---|---|---|
| | **Marginal** | **Conditional** |
| ***r²*** | .48 | .88 |
| **ICC (Participant)** | | .77 |

The response vitality variable $\gamma_i$ is modelled as:

$$\gamma(\text{vitality})_i = \beta_0 + \beta_1 * time_i + \beta_2 * group_i + \beta_3 * (time_i * group_i) + \beta_4 * ROF_i + \beta_5 * (time_i * ROF_i) + \beta_6 * (group_i * ROF_i) + \beta_7 * TC_i + \beta_8 * (time_i * group_i * TC_i) + \beta_{10} * TS_i + \beta_{11} * (time_i * TS_i) + \beta_{12} * (group_i * TS_i) + u_{i0} + \epsilon_i$$

- $\gamma_i$: response for the *i*-th observation.
- $\beta$: fixed intercept
- $u_{i0}$: random intercept for the *i*-th participant.
- $\epsilon_i$: residual error term for the *i*-th observation.
- The random effects structure: $u_{i2} \sim N(0, \sigma^2)$

*Note*. All subjective variables (vitality, ROF, TS, TC) were z-standardised prior to the analysis. The β-values represent the standardized fixed effects predicting ROF. Random intercepts were specified for participants. Bootstrapping with 1000 iterations for all values was used. Results reaching the significance threshold of $p < .05$ were marked in bold.

Supplementary Table 3

Balloon Analogue Risk Task (BART) Mixed Model comparison and the model building/selection process

| **Model** | **Nested / simpler Model** | **Random effects** | **Fixed effects** | **Model fit** | | | | | **LRT against nested** | |
|---|---|---|---|---|---|---|---|---|---|---|
| | | | | **AIC** | **BIC** | **LL** | **df** | **deviance** | **X²** | ***p*** |
| Separate interaction | - | Intercept participants | Timepoint x group + fatigue + fatigue x time + fatigue x group + vitality + vitality x time + vitality x group + TC + TC x time + TC x group + TS + TS x time + TS x group + SUPPS-P x sens seeking x risk | -59.01 | 32.19 | 63.50 | - | -127.01 | - | - |
| Separate interaction + triple interaction fatigue | Separate interaction | Intercept participants | Timepoint x group + fatigue + fatigue x time x group + vitality + vitality x time + vitality x group + TC + TC x time + TC x group + TS + TS x time + TS x group + SUPPS-P x sens seeking x risk | -55.64 | 46.28 | 65.82 | 4 | -131.64 | 4.63 | .33 |
| Separate interaction + triple interaction vitality | Separate interaction | Intercept participants | Timepoint x group + fatigue + fatigue x time + fatigue x group + vitality + vitality x time x group + TC + TC x time + TC x group + TS + TS x time + TS x group + SUPPS-P x sens seeking x risk | -61.11 | 40.81 | 68.55 | 4 | -137.11 | 10.10 | **.038** |
| Separate interaction + triple interaction TC | Separate interaction + triple interaction vitality | Intercept participants | Timepoint x group + fatigue + fatigue x time + fatigue x group + vitality + vitality x time + vitality x group + TC + TC x time x group + TS + TS x time + TS x group + SUPPS-P x sens seeking x risk | -57.44 | 44.49 | 66.72 | 0 | -133.44 | 0 | - |

| | | | | | | | | | | |
|---|---|---|---|---|---|---|---|---|---|---|
| Separate interaction + triple interaction TS | Separate interaction + triple interaction vitality | Intercept participants | Timepoint x group + fatigue + fatigue x time + fatigue x group + vitality + vitality x time + vitality x group + TC + TC x time + TC x group + TS + TS x time x group + SUPPS-P x sens seeking x risk | -65.45 | 36.47 | 70.73 | 0 | -141.45 | 0 | - |
| Separate interaction + triple interaction vitality & TS | Separate interaction + triple interaction TS | Intercept participants | Timepoint x group + fatigue + fatigue x time + fatigue x group + vitality + vitality x time x group + TC + TC x time + TC x group + TS + TS x time x group + SUPPS-P x sens seeking x risk | -59.64 | 53.01 | 71.82 | 4 | -143.64 | 2.19 | .70 |
| Triple interaction | Separate interaction + triple interaction TS | Intercept participants | Timepoint x group + fatigue + fatigue x time x group + vitality + vitality x time x group + TC + TC x time x group + TS + TS x time x group + SUPPS-P x sens seeking x risk | -51.15 | 82.96 | 75.57 | 12 | -151.15 | 9.69 | .64 |

| **BART final model:** Separate interaction + triple interaction TS | | | | | |
|---|---|---|---|---|---|
| | **Fixed Effects** | | | | |
| **Model parameter** | **Estimate/*β*** | ***SE*** | **95% CI** | ***t*** | ***p*** |
| Intercept | 2.56 | 0.39 | 1.79 – 3.33 | 6.61 | **<.001** |
| T2 | 0.01 | 0.04 | -0.07 – 0.08 | 0.23 | .816 |
| T3 | -0.02 | 0.04 | -0.11 – 0.06 | -0.57 | .571 |
| Group PHS | 0.00 | 0.13 | -0.26 – 0.26 | 0.00 | .997 |
| Group CHS | -0.07 | 0.12 | -0.32 – 0.17 | -0.60 | .552 |
| Group PHS X T2 | 0.13 | 0.09 | -0.05 – 0.31 | 1.43 | .158 |
| Group PHS X T3 | 0.06 | 0.06 | -0.07 – 0.19 | 0.93 | .355 |
| Group CHS X T2 | 0.01 | 0.06 | -0.11 – 0.14 | 0.24 | .808 |
| Group CHS X T3 | 0.17 | 0.07 | 0.02 – 0.31 | 2.32 | **.023** |
| SUPPS-P | 0.23 | 0.16 | -0.08 – 0.54 | 1.51 | .135 |
| Sensation seeking | 0.06 | 0.08 | -0.10 – 0.22 | 0.79 | .433 |
| Risk-taking | -0.04 | 0.04 | -0.11 – 0.04 | -0.96 | .342 |
| Vitality | 0.02 | 0.04 | -0.06 – 0.10 | 0.48 | .629 |
| Group PHS X Vitality | -0.03 | 0.06 | -0.14 – 0.08 | -0.54 | .593 |
| Group CHS X Vitality | 0.03 | 0.06 | -0.09 – 0.16 | 0.53 | .597 |
| Vitality X T2 | -0.00 | 0.03 | -0.07 – 0.07 | -0.05 | .963 |
| Vitality X T3 | -0.09 | 0.04 | -0.16 – -0.02 | -2.55 | **.013** |
| ROF | 0.04 | 0.03 | -0.02 – 0.09 | 1.41 | .162 |
| Group PHS X ROF | -0.05 | 0.05 | -0.15 – 0.05 | -0.95 | .348 |
| Group CHS X ROF | 0.01 | 0.05 | -0.09 – 0.10 | 0.15 | .880 |

| | | | | | |
|---|---|---|---|---|---|
| ROF X T2 | -0.04 | 0.03 | -0.11 – 0.02 | -1.25 | .215 |
| ROF X T3 | -0.10 | 0.04 | -0.17 – -0.02 | -2.54 | **.013** |
| TC | -0.00 | 0.04 | -0.08 – 0.08 | -0.01 | .993 |
| Group PHS X TC | 0.01 | 0.04 | -0.07 – 0.09 | 0.23 | .816 |
| Group CHS X TC | 0.02 | 0.05 | -0.08 – 0.11 | 0.32 | .747 |
| TC X T2 | -0.05 | 0.03 | -0.11 – 0.02 | -1.51 | .135 |
| TC X T3 | 0.02 | 0.04 | -0.05 – 0.09 | 0.64 | .526 |
| TS | 0.04 | 0.03 | -0.03 – 0.11 | 1.26 | .213 |
| Group PHS X TS | 0.01 | 0.06 | -0.11 – 0.13 | 0.10 | .923 |
| Group CHS X TS | -0.11 | 0.05 | -0.21 – -0.02 | -2.35 | **.022** |
| TS X T2 | 0.03 | 0.05 | -0.07 – 0.12 | 0.55 | .583 |
| TS X T3 | -0.02 | 0.04 | -0.11 – 0.07 | -0.52 | .607 |
| Group PHS X TS X T2 | -0.27 | 0.08 | -0.42 – -0.11 | -3.32 | **.001** |
| Group PHS X TS X T3 | -0.07 | 0.06 | -0.20 – 0.06 | -1.11 | .271 |
| Group CHS X TS X T2 | 0.01 | 0.07 | -0.12 – 0.15 | 0.18 | .861 |
| Group CHS X TS X T3 | 0.04 | 0.06 | -0.08 – 0.16 | 0.69 | .491 |

| | **Random Effects** | |
|---|---|---|
| | **Variance** | ***SD*** |
| Participant (Intercept) | 0.06 | 0.24 |
| Residual | 0.004 | 0.07 |

| | **Model fit** | |
|---|---|---|
| | **Marginal** | **Conditional** |
| ***r²*** | .13 | .93 |

| ICC (Participant) | .92 |
|---|---|

The response variable adjusted average pumps (AAP) $\gamma_i$ is modelled as:

$$\gamma(\log(\text{AAP}))_i = \beta_0 + \beta_1 * time_i + \beta_2 * group_i + \beta_3 * (time_i * group_i) + \beta_4 * SUPPSP_i + \beta_5 * SensSeeking_i + \beta_6 * Risk + \beta_7 * ROF_i + \beta_8 * (time_i * ROF_i) + \beta_9 * (group_i * ROF_i) + \beta_{10} * vitality_i + \beta_{11} * (time_i * vitality_i) + \beta_{12} * (group_i * vitality_i) + \beta_{13} * TC_i + \beta_{14} * (time_i * TC_i) + \beta_{15} * (group_i * TC_i) + \beta_{16} * TS_i + \beta_{17} * (time_i * group_i * TS_i) + u_{i0} + \epsilon_i$$

- $\gamma_i$: response for the *i*-th observation.
- $\beta$: fixed intercept
- $u_{i0}$: random intercept for the *i*-th participant.
- $\epsilon_i$: residual error term for the *i*-th observation.
- The random effects structure: $u_{i2} \sim N(0, \sigma^2)$

*Note*. All subjective variables (vitality, ROF, TS, TC) were z-standardised prior to the analysis. The β-values represent the standardized fixed effects predicting ROF. Random intercepts were specified for participants. Bootstrapping with 1000 iterations for all values was used. Results reaching the significance threshold of $p < .05$ were marked in bold.

Supplementary Table 4

Beads Task (BT) Mixed Model comparison and the model building/selection process

| **Model** | **Nested / simpler Model** | **Random effects** | **Fixed effects** | **Model fit** | | | | | **LRT against nested** | |
|---|---|---|---|---|---|---|---|---|---|---|
| | | | | **AIC** | **BIC** | **LL** | **df** | **deviance** | **X²** | ***p*** |
| Separate interaction | - | Intercept participants | Timepoint x group + fatigue + fatigue x time + fatigue x group + vitality + vitality x time + vitality x group + TC + TC x time + TC x group + TS + TS x time + TS x group + SUPPS-P x sens seeking x risk | 59.62 | 150.82 | 4.18 | - | -8.38 | - | - |
| Separate interaction + triple interaction fatigue | Separate interaction | Intercept participants | Timepoint x group + fatigue + fatigue x time x group + vitality + vitality x time + vitality x group + TC + TC x time + TC x group + TS + TS x time + TS x group + SUPPS-P x sens seeking x risk | 62.51 | 164.43 | -13.49 | 4 | -13.49 | 5.11 | .28 |
| Separate interaction + triple interaction vitality | Separate interaction | Intercept participants | Timepoint x group + fatigue + fatigue x time + fatigue x group + vitality + vitality x time x group + TC + TC x time + TC x group + TS + TS x time + TS x group + SUPPS-P x sens seeking x risk | 64.42 | 166.34 | 5.79 | 4 | -11.58 | 3.20 | .52 |
| Separate interaction + triple interaction TC | Separate interaction | Intercept participants | Timepoint x group + fatigue + fatigue x time + fatigue x group + vitality + vitality x time + vitality x group + TC + TC x time x group + TS + TS x time + TS x group + SUPPS-P x sens seeking x risk | 63.75 | 165.67 | 6.13 | 4 | -12.25 | 3.88 | .42 |

| | | | | | | | | | | |
|---|---|---|---|---|---|---|---|---|---|---|
| Separate interaction + triple interaction TS | Separate interaction | Intercept participants | Timepoint x group + fatigue + fatigue x time + fatigue x group + vitality + vitality x time + vitality x group + TC + TC x time + TC x group + TS + TS x time x group + SUPPS-P x sens seeking x risk | 64.31 | 166.23 | 5.84 | 4 | -11.69 | 3.31 | .51 |
| Triple interaction | Separate interaction | Intercept participants | Timepoint x group + fatigue + fatigue x time x group + vitality + vitality x time x group + TC + TC x time x group + TS + TS x time x group + SUPPS-P x sens seeking x risk | 77.65 | 211.76 | 11.17 | 16 | -22.35 | 13.97 | .60 |

| BT final model: Separate interaction | | | | | |
|---|---|---|---|---|---|
| | Fixed Effects | | | | |
| **Model parameter** | **Estimate/β** | ***SE*** | **95% CI** | ***t*** | ***p*** |
| Intercept | 2.72 | 0.63 | 1.47 – 3.97 | 4.33 | **<.001** |
| T2 | -0.01 | 0.07 | -0.14 – 0.13 | -0.13 | .898 |
| T3 | 0.02 | 0.07 | -0.12 – 0.16 | 0.32 | .748 |
| Group PHS | -0.21 | 0.22 | -0.64 – 0.22 | -0.97 | .337 |
| Group CHS | -0.11 | 0.20 | -0.52 – 0.29 | -0.55 | .585 |
| Group PHS X T2 | 0.02 | 0.15 | -0.28 – 0.32 | 0.13 | .894 |
| Group PHS X T3 | -0.05 | 0.11 | -0.27 – 0.17 | -0.46 | .645 |
| Group CHS X T2 | 0.01 | 0.12 | -0.22 – 0.25 | 0.11 | .910 |
| Group CHS X T3 | -0.00 | 0.14 | -0.28 – 0.27 | -0.02 | .986 |
| SUPPS-P | -0.32 | 0.25 | -0.82 – 0.18 | -1.26 | .211 |
| Sensation seeking | 0.08 | 0.13 | -0.18 – 0.34 | 0.62 | .540 |
| Risk-taking | -0.04 | 0.06 | -0.17 – 0.08 | -0.71 | .479 |
| Vitality | 0.07 | 0.08 | -0.08 – 0.23 | 0.91 | .368 |
| Group PHS X Vitality | 0.02 | 0.10 | -0.19 – 0.23 | 0.20 | .840 |
| Group CHS X Vitality | -0.14 | 0.12 | -0.37 – 0.09 | -1.19 | .237 |
| Vitality X T2 | 0.02 | 0.07 | -0.12 – 0.15 | 0.26 | .797 |
| Vitality X T3 | -0.03 | 0.07 | -0.16 – 0.11 | -0.38 | .708 |
| ROF | -0.00 | 0.05 | -0.10 – 0.10 | -0.06 | .950 |
| Group PHS X ROF | -0.15 | 0.10 | -0.35 – 0.05 | -1.48 | .143 |
| Group CHS X ROF | -0.04 | 0.09 | -0.21 – 0.14 | -0.44 | .665 |

| | | | | | |
|---|---|---|---|---|---|
| ROF X T2 | -0.01 | 0.06 | -0.13 – 0.11 | -0.20 | .845 |
| ROF X T3 | -0.01 | 0.07 | -0.15 – 0.14 | -0.11 | .910 |
| TC | 0.01 | 0.08 | -0.15 – 0.16 | 0.09 | .926 |
| Group PHS X TC | -0.21 | 0.08 | -0.37 – -0.05 | -2.59 | **.012** |
| Group CHS X TC | 0.01 | 0.09 | -0.17 – 0.18 | 0.08 | .936 |
| TC X T2 | -0.01 | 0.06 | -0.13 – 0.12 | -0.13 | .896 |
| TC X T3 | -0.05 | 0.07 | -0.18 – 0.09 | -0.69 | .492 |
| TS | -0.03 | 0.07 | -0.17 – 0.10 | -0.49 | .626 |
| Group PHS X TS | -0.16 | 0.10 | -0.36 – 0.04 | -1.60 | .113 |
| Group CHS X TS | 0.01 | 0.08 | -0.16 – 0.18 | 0.12 | .909 |
| TS X T2 | 0.07 | 0.06 | -0.04 – 0.19 | 1.27 | .210 |
| TS X T3 | 0.03 | 0.05 | -0.08 – 0.13 | 0.55 | .583 |

| | **Random Effects** | |
|---|---|---|
| | **Variance** | ***SD*** |
| **Participant (Intercept)** | 0.15 | 0.38 |
| **Residual** | 0.019 | 0.14 |

| | **Model fit** | |
|---|---|---|
| | **Marginal** | **Conditional** |
| ***r²*** | 0.18 | .91 |
| **ICC (Participant)** | | .89 |

The response variable draws to decision (DTD) $\gamma_i$ is modelled as:

$$\gamma(\log(\mathrm{DTD}))_i = \beta_0 + \beta_1 * time_i + \beta_2 * group_i + \beta_3 * (time_i * group_i) + \beta_4 * SUPPSP_i + \beta_5 * SensSeeking_i + \beta_6 * Risk + \beta_7 * ROF_i + \beta_8 * (time_i * ROF_i) + \beta_9 * (group_i * ROF_i) + \beta_{10} * vitality_i + \beta_{11} * (time_i * vitality_i) + \beta_{12} * (group_i * vitality_i) + \beta_{13} * TC_i + \beta_{14} * (time_i * TC_i) + \beta_{15} * (group_i * TC_i) + \beta_{16} * TS_i + \beta_{17} * (time_i * TS_i) + \beta_{18} * (group_i * TS_i) + u_{i0} + \epsilon_i$$

- $\gamma_i$: response for the *i*-th observation.
- $\beta$: fixed intercept
- $u_{i0}$: random intercept for the *i*-th participant.
- $\epsilon_i$: residual error term for the *i*-th observation.
- The random effects structure is represented by: $u_{i2} \sim N(0, \sigma^2)$

*Note*. All subjective variables (vitality, ROF, TS, TC) were z-standardised prior to the analysis. The β-values represent the standardized fixed effects predicting ROF. Random intercepts were specified for participants. Bootstrapping with 1000 iterations for all values was used. Results reaching the significance threshold of $p < .05$ were marked in bold.

**APPENDIX FIGURE 1**

Interaction effect fatigue - time x group x vitality

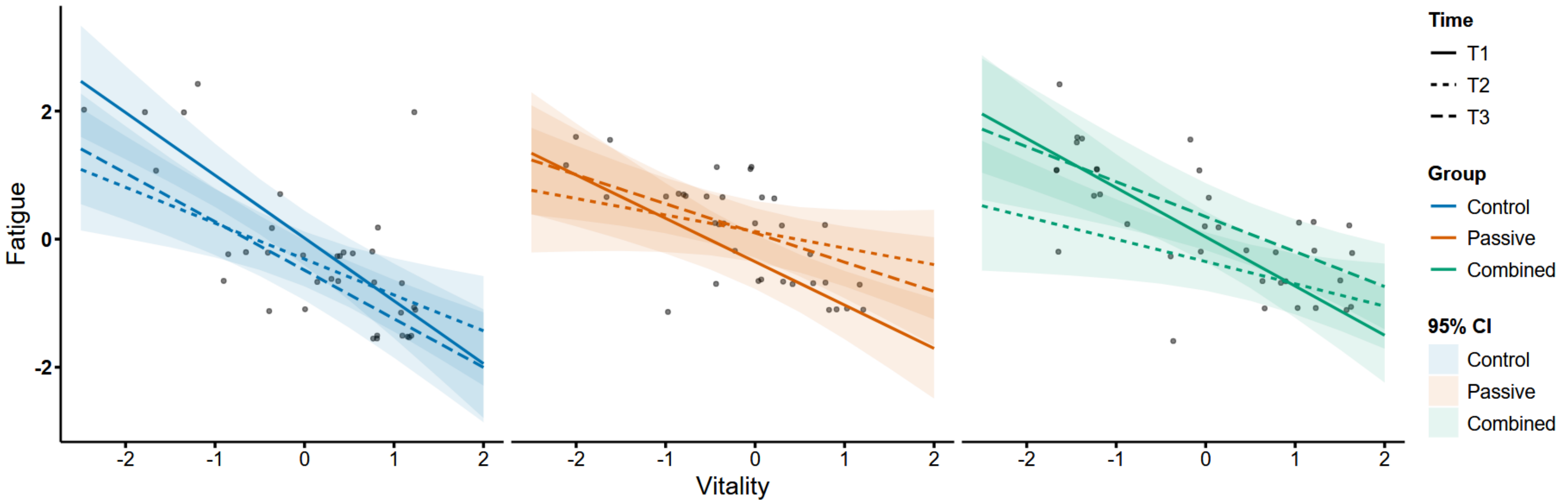


*Note*. Linear mixed model results for subjective perception of fatigue. Graphics show the fixed effects as the coloured bold line for fatigue interacting with vitality, with 95% confidence intervals (CI) represented in the shaded areas. Respectively, the difference between control, passive and combined group is illustrated, as well as the time (T1, pre intervention; T2, during intervention; T3, post intervention). The grey points represent individuals of the groups over time.

## APPENDIX FIGURE 2

Interaction effect vitality - time x group x fatigue

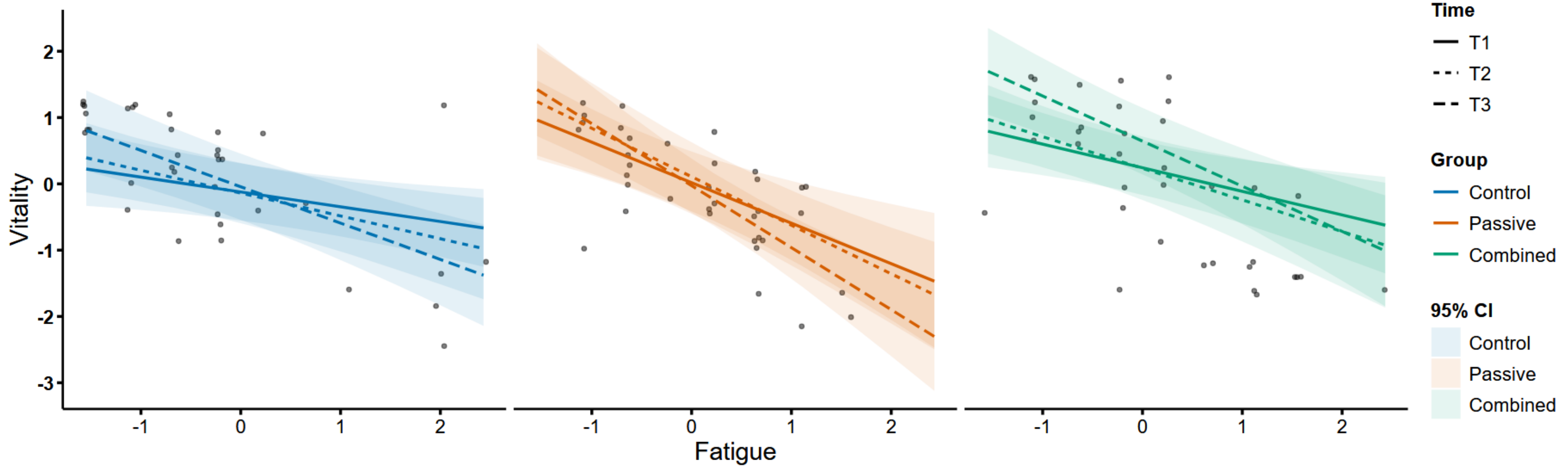


*Note*. Linear mixed model results for subjective perception of vitality. Graphics show the fixed effects as the coloured bold line for vitality interacting with fatigue, with 95% confidence intervals (CI) represented in the shaded areas. Respectively, the difference between control, passive and combined group is illustrated, as well as the time (T1, pre intervention; T2, during intervention; T3, post intervention). The grey points represent individuals of the groups over time.

**APPENDIX FIGURE 3**

Interaction effect vitality - time x group x TC

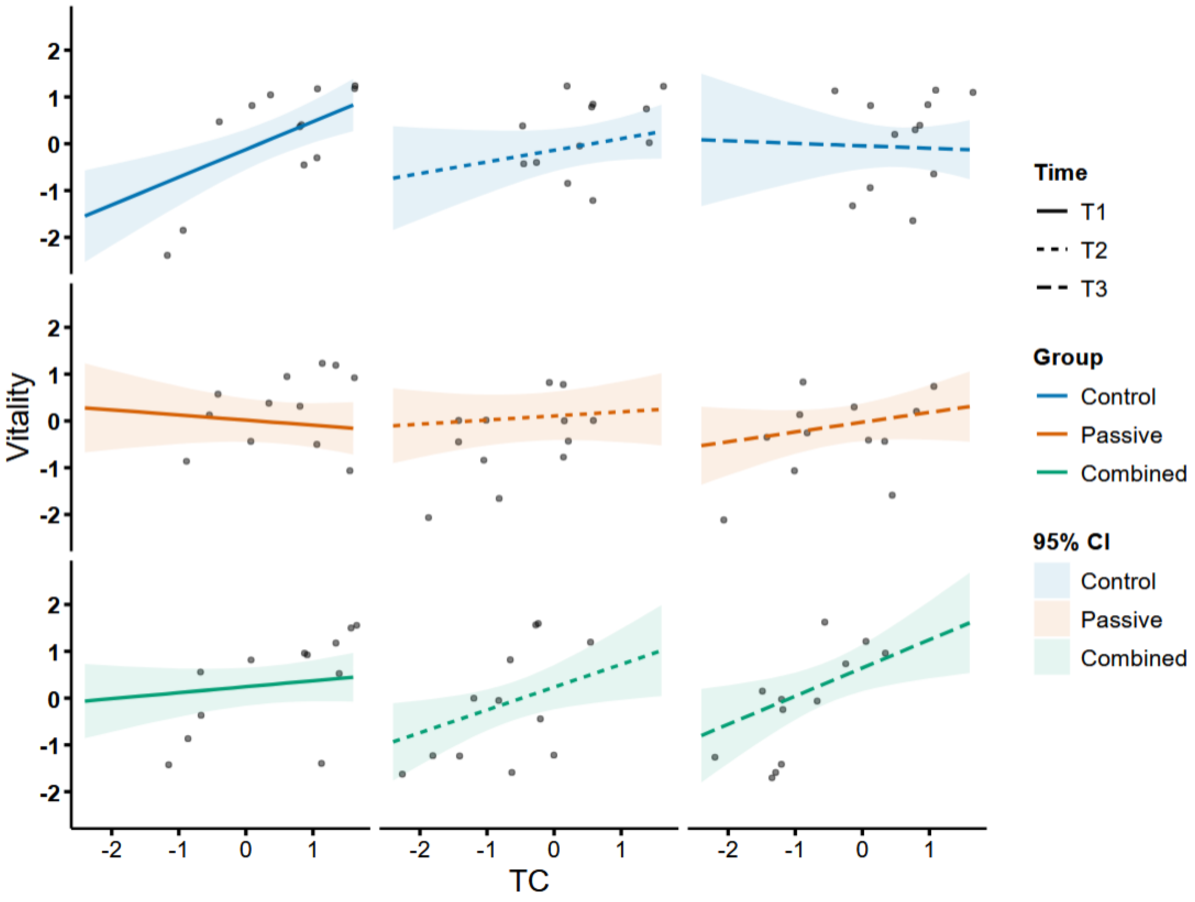


*Note*. Linear mixed model results for subjective perception of vitality. Graphics show the fixed effects as the coloured bold line for vitality interacting with thermal comfort (TC), with 95% confidence intervals (CI) represented in the shaded areas. Respectively, the difference between control, passive and combined group is illustrated, as well as the time (T1, pre intervention; T2, during intervention; T3, post intervention). The grey points represent individuals of the groups over time.

## APPENDIX FIGURE 4

Interaction effect risk-taking behaviour - time x group x TS

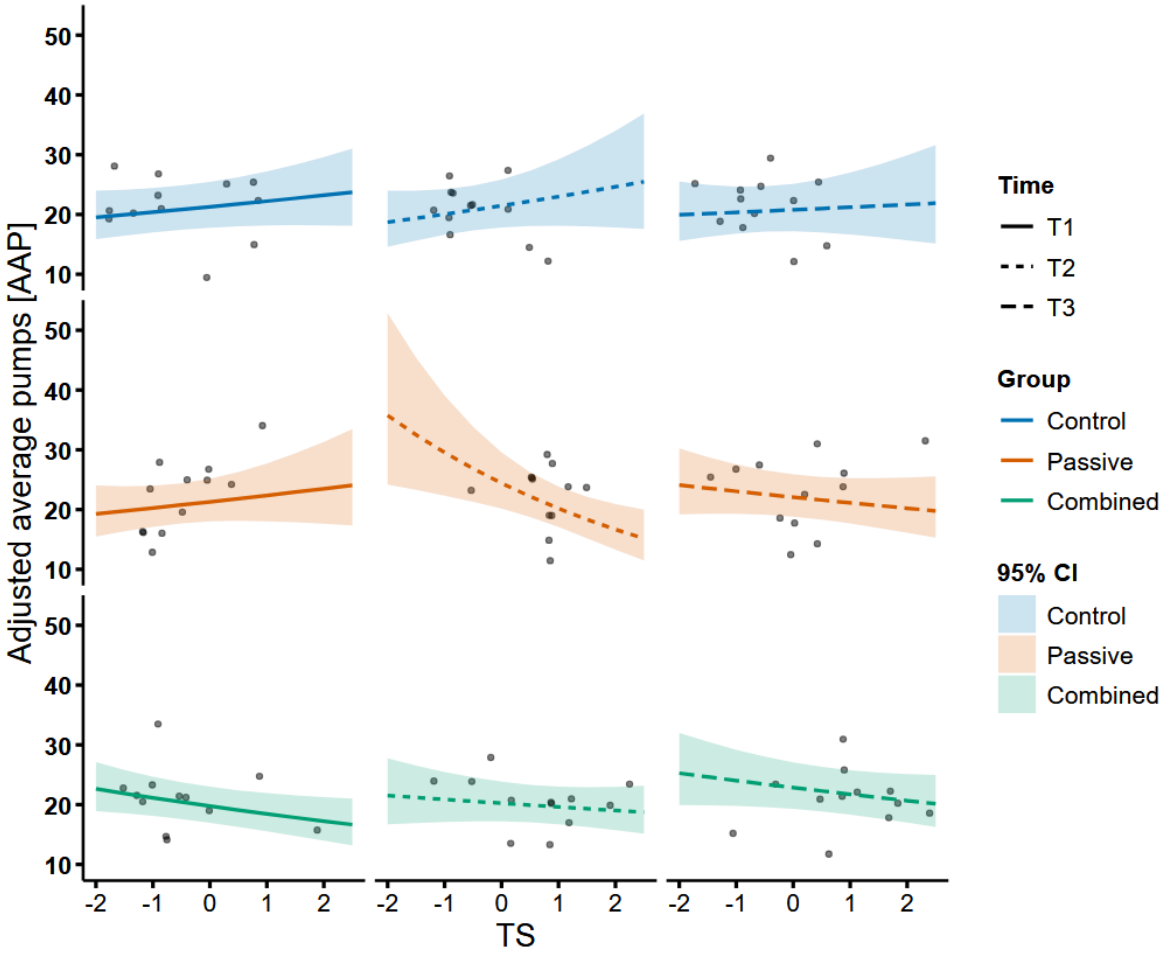


*Note*. Linear mixed model results for risk-taking behaviour. The Balloon Analogue Risk Task (BART) measured risk-taking behaviour in adjusted average pumps (AAP). Graphics show the fixed effects as the coloured bold line for risk-taking interacting with thermal sensation (TS), with 95% confidence intervals (CI) represented in the shaded areas. Respectively, the difference between control, passive and combined group is illustrated, as well as the time (T1, pre intervention; T2, during intervention; T3, post intervention). The grey points represent individuals of the groups over time.

## APPENDIX FIGURE 5

Interaction effect risk-taking behaviour - time x group x fatigue

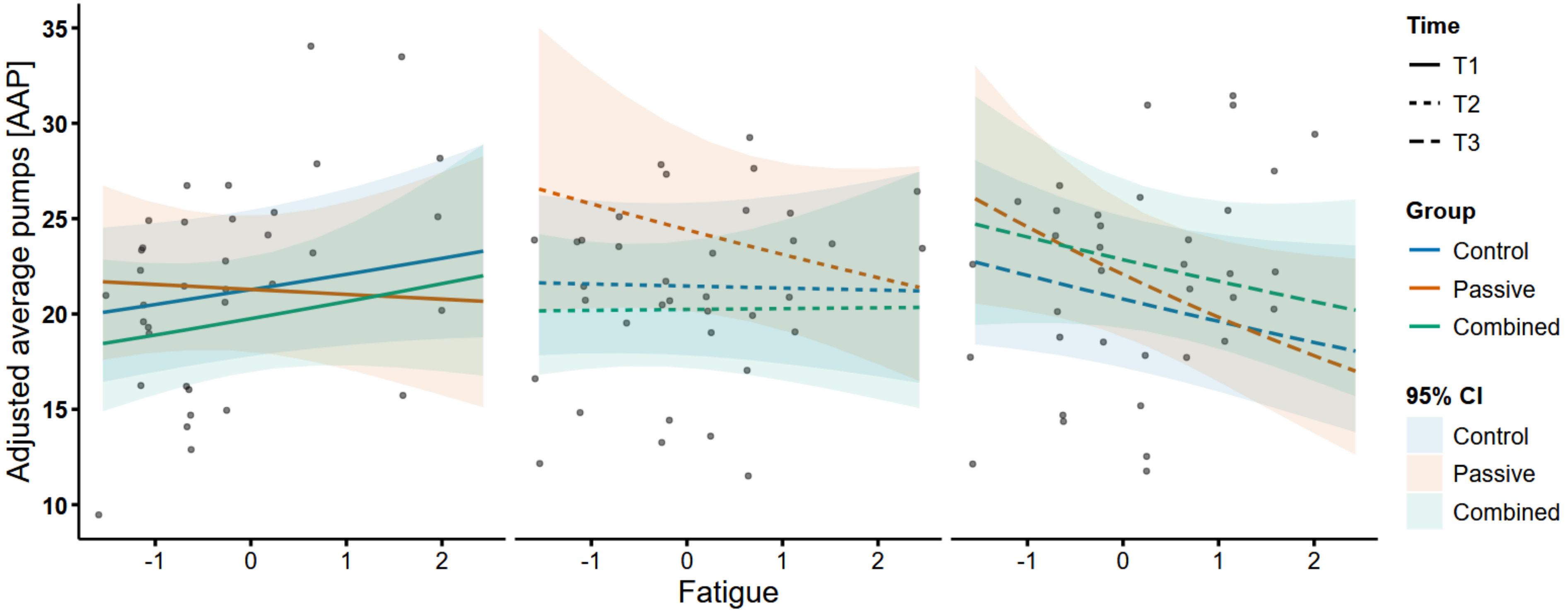


*Note*. Linear mixed model results for risk-taking behaviour. The Balloon Analogue Risk Task (BART) measured risk-taking behaviour in adjusted average pumps (AAP). Graphics show the fixed effects as the coloured bold line for risk-taking interacting with fatigue, with 95% confidence intervals (CI) represented in the shaded areas. Respectively, the difference between control, passive and combined group is illustrated, as well as the time (T1, pre intervention; T2, during intervention; T3, post intervention). The grey points represent individuals of the groups over time.

**APPENDIX FIGURE 6**

Interaction effect risk-taking behaviour - time x group x vitality

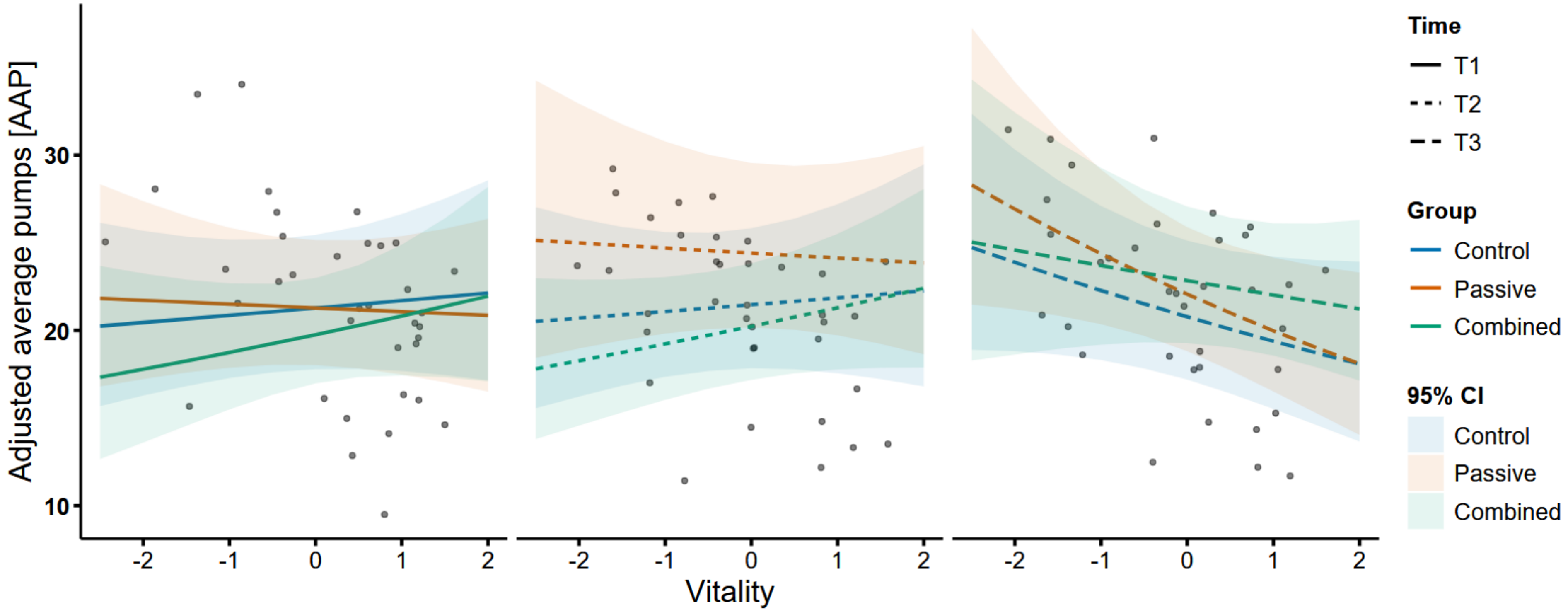


*Note*. Linear mixed model results for risk-taking behaviour. The Balloon Analogue Risk Task (BART) measured risk-taking behaviour in adjusted average pumps (AAP). Graphics show the fixed effects as the coloured bold line for risk-taking interacting with vitality, with 95% confidence intervals (CI) represented in the shaded areas. Respectively, the difference between control, passive and combined group is illustrated, as well as the time (T1, pre intervention; T2, during intervention; T3, post intervention). The grey points represent individuals of the groups over time.

## APPENDIX FIGURE 7

Interaction effect reflection impulsivity - time x group x TC

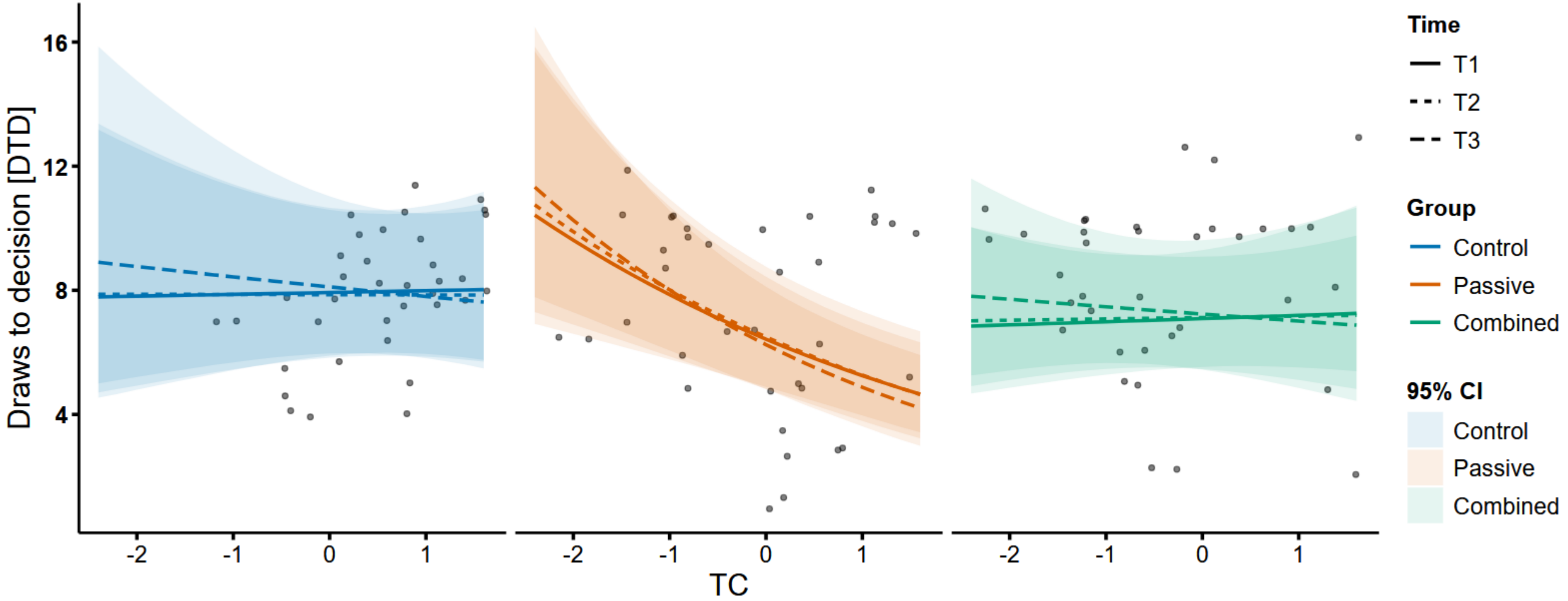


*Note*. Linear mixed model results for reflection impulsivity. The Beads Taks (BT) measured reflection impulsivity in draws to decision (DTD). Graphics show the fixed effects as the coloured bold line for risk-taking interacting with vitality, with 95% confidence intervals (CI) represented in the shaded areas. Respectively, the difference between control, passive and combined group is illustrated, as well as the time (T1, pre intervention; T2, during intervention; T3, post intervention). The grey points represent individuals of the groups over time.
